# Comprehensive characterization of amino acid positions in protein structures reveals molecular effect of missense variants

**DOI:** 10.1073/pnas.2002660117

**Published:** 2020-10-26

**Authors:** Sumaiya Iqbal, Eduardo Pérez-Palma, Jakob B. Jespersen, Patrick May, David Hoksza, Henrike O. Heyne, Shehab S. Ahmed, Zaara T. Rifat, M. Sohel Rahman, Kasper Lage, Aarno Palotie, Jeffrey R. Cottrell, Florence F. Wagner, Mark J. Daly, Arthur J. Campbell, Dennis Lal

**Affiliations:** ^a^Center for the Development of Therapeutics, Broad Institute of MIT and Harvard, Cambridge, MA 02142;; ^b^Stanley Center for Psychiatric Research, Broad Institute of MIT and Harvard, Cambridge, MA, 02142;; ^c^Program in Medical and Population Genetics, Broad Institute of MIT and Harvard, Cambridge, MA 02142;; ^d^Analytic and Translational Genetics Unit, Massachusetts General Hospital, Boston, MA 02114;; ^e^Genomic Medicine Institute, Lerner Research Institute, Cleveland Clinic, Cleveland, OH 44195;; ^f^Department of Bio and Health Informatics, Technical University of Denmark, 2800 Kgs. Lyngby, Denmark;; ^g^Luxembourg Centre for Systems Biomedicine, University of Luxembourg, 4365 Esch-sur-Alzette, Luxembourg;; ^h^Department of Software Engineering, Faculty of Mathematics and Physics, Charles University, Prague 11636, Czech Republic;; ^i^Institute for Molecular Medicine Finland, University of Helsinki, 00100 Helsinki, Finland;; ^j^Computer Science and Engineering, Bangladesh University of Engineering and Technology, Dhaka-1205, Bangladesh;; ^k^Department of Surgery, Massachusetts General Hospital, Boston, MA 02114;; ^l^Cologne Center for Genomics, University of Cologne, 50931 Cologne, Germany;; ^m^Epilepsy Center, Neurological Institute, Cleveland Clinic, Cleveland, OH 44195

**Keywords:** missense variant interpretation, protein structure and function, disease variation effect, 3D mutational hotspot, machine learning

## Abstract

Interpretation of the colossal number of genetic variants identified from sequencing applications is one of the major bottlenecks in clinical genetics, with the inference of the effect of amino acid-substituting missense variations on protein structure and function being especially challenging. Here we characterize the three-dimensional (3D) amino acid positions affected in pathogenic and population variants from 1,330 disease-associated genes using over 14,000 experimentally solved human protein structures. By measuring the statistical burden of variations (i.e., point mutations) from all genes on 40 3D protein features, accounting for the structural, chemical, and functional context of the variations’ positions, we identify features that are generally associated with pathogenic and population missense variants. We then perform the same amino acid-level analysis individually for 24 protein functional classes, which reveals unique characteristics of the positions of the altered amino acids: We observe up to 46% divergence of the class-specific features from the general characteristics obtained by the analysis on all genes, which is consistent with the structural diversity of essential regions across different protein classes. We demonstrate that the function-specific 3D features of the variants match the readouts of mutagenesis experiments for BRCA1 and PTEN, and positively correlate with an independent set of clinically interpreted pathogenic and benign missense variants. Finally, we make our results available through a web server to foster accessibility and downstream research. Our findings represent a crucial step toward translational genetics, from highlighting the impact of mutations on protein structure to rationalizing the variants’ pathogenicity in terms of the perturbed molecular mechanisms.

## Significance

Recent large-scale sequencing efforts have enabled the detection of millions of missense variants. Elucidating their functional effect is of crucial importance but challenging. We approach this problem by performing a wide-scale characterization of missense variants from 1,330 disease-associated genes using >14,000 protein structures. We identify 3D features associated with pathogenic and benign variants that unveiled the mutations’ effect at the molecular level. We further extend our analysis to account for the different essential structural regions in proteins performing different functions. By analyzing variants from 24 gene groups encoding for different protein functional families, we capture function-specific characteristics of missense variants, which match the experimental readouts. We show that our results derived using structural data will effectively inform variant interpretation.

Genetic screening is increasingly applied in clinical practice, especially for the diagnosis of rare monogenic diseases and cancer, leading to the identification of a rapidly growing number of genetic variations (1, 2). Most of these are missense variations, which cause an amino acid substitution upon a single nucleotide change in the protein-coding region of the genome. Detection of such missense variations by high-throughput DNA sequencing is now relatively straightforward. Predicting their association with disease from sequencing output alone, instead, remains challenging because missense variations can be either benign or pathogenic, and both types coexist in almost every disease-associated gene (3). To discover how a missense variant is implicated in a disease requires knowledge of the consequence of the amino acid substitution (i.e., variation) on the protein structure and function. A plethora of disease-associated (“pathogenic”) and benign (“population”) variants has been collected in multiple databases such as Online Mendelian Inheritance in Man (OMIM) (4), Human Gene Mutation Database (HGMD) (5), ClinVar (6), Exome Aggregation Consortium (ExAC) (3), and Genome Aggregation Database (gnomAD) (7). These resources, along with an increasing amount of protein structure data available in the Protein Data Bank (PDB) (8), now offer an unprecedented opportunity to characterize pathogenic and benign missense variants in the context of protein structure–function relationships. Progress in this direction can aid variant interpretation, inform experiments, and help accelerate personalized drug discovery.

Current in silico methods for variant pathogenicity prediction employ a variety of machine learning algorithms, which are trained on pathogenic and population variant data using many features such as evolutionary information (“conserved sites”), gene-level properties (e.g., “essentiality”), and specific amino acid exchanges in protein sequences (9–12). Although the ability to predict pathogenicity is improving (13, 14), the output scores of the predictors do not advance our knowledge about the molecular pathology of the associated disorder. Since a computational “black box” model generates these scores, they are not biologically interpretable; that is, it is not possible to understand why a particular missense variant is predicted to have a high or low pathogenicity score or to establish what the molecular effect of the variation will be.

Biological insights into the effect of pathogenic missense variants can reportedly be gained by analyzing the relationship between point mutations and protein structures (15–18). Several studies have shown that the damaging consequences of missense variations are linked to the properties (19–21) and localization of the altered amino acid residues in the protein structure (22–26). Subsequently, resources have been developed to predict and report the impact of amino acid substitutions on protein structures: missense3D (27) predicts the changes in structure and free energy upon mutations, which is applicable to both experimental structures and homology models of the structures; SuSPect (28) predicts the association between missense variations and their phenotypic impact leveraging information of protein–protein interaction networks; VarSite (29) presents a range of features associated with the variants (related diseases, structural annotations, pathways, tissue specificity, etc.).

The variant interpretation guidelines proposed by American College of Medical Genetics and Genomics (ACMG) list the presence of an amino acid substitution in mutational hotspots (PM1 criterion (30), i.e., sites displaying frequent occurrence of pathogenic mutations and depleted in benign variants) as moderate evidence for pathogenicity. Such hotspots can be located in any “functional domain,” namely, a region of the protein known to be critical for function, but also in “less well-characterized regions.” Indeed, because proteins are molecules characterized by a dense network of both intramolecular and intermolecular interactions, amino acid substitutions occurring in many different positions can have far-reaching consequences on protein structure and stability (31). Further, proteins performing a similar function often have conserved structural regions that are intolerant to substitution, and such regions vary for proteins that carry out different functions (32, 33). For example, the “voltage-sensing” helical regions of the ion transporters (e.g., sodium channel family) are predominantly enriched with pathogenic missense variations, causing several forms of neurological channelopathies (34–36). Similarly, in certain enzymes (kinase, phosphatase, etc.), mutations of distinctive catalytic and regulatory sites in the structure are shown to be associated with diverse phenotypes (37, 38).

With this study, we sought to bridge the gap between genetic variation data and molecular phenotype through the analysis of features of single amino acids in the context of the native three-dimensional (3D) protein structure (“3D sites”). The rationale behind this approach is that features of the 3D sites (“3D features”) that are more frequently mutated in pathogenic variants than in benign variants (“3D mutational hotspot”) are likely to be important for protein fitness, and therefore could contribute to explaining the molecular determinants of pathogenicity. Concomitantly, we speculate that knowledge of specific features of 3D mutational hotspots for individual protein functional classes (e.g., kinases, transporters, cytoskeletal proteins) can considerably help with formulating informed hypotheses in the interpretation of variant pathogenicity.

## Results

**Fig. 1.**
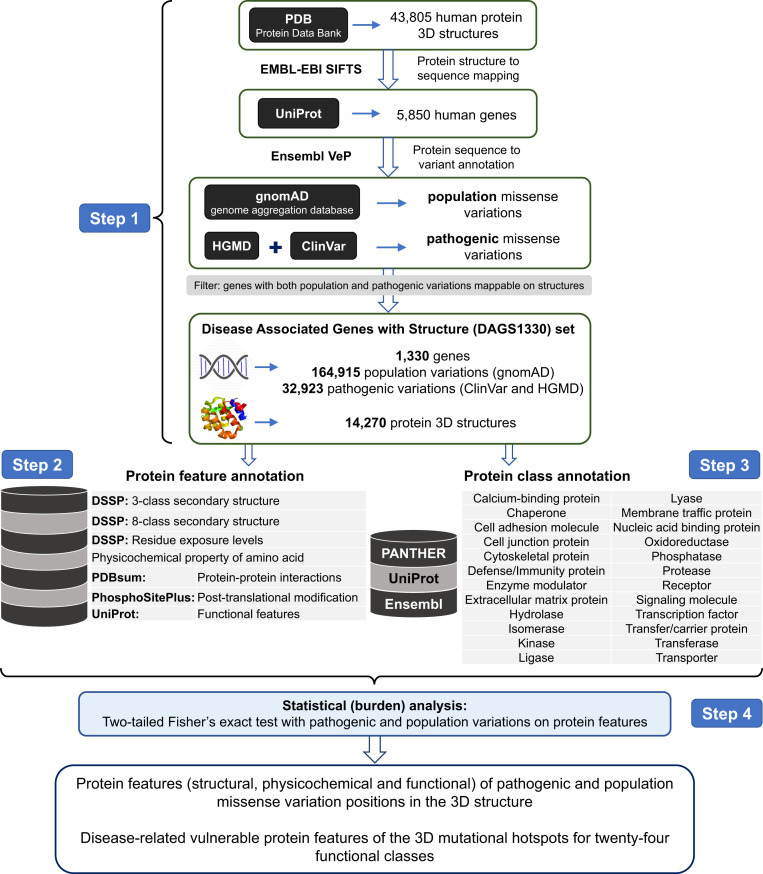
Illustration of the study design and objectives. Step 1: Dataset preparation and missense variant to protein structure mapping. Experimentally solved human protein structures are collected from the PDB (8) (in January 2018) and mapped to UniProt-defined canonical protein sequences using the SIFTS database (39). The missense variants are assembled from three databases: general population variants from gnomAD (public release 2.0.2), disease mutations from HGMD (professional release 2018.4 and 2019.2), and pathogenic and likely pathogenic variants from ClinVar (February 2018 and 2019 releases). Finally, the analysis is restricted to the 1,330 genes (DAGS1330 set) for which both population (*n* = 164,915) and pathogenic (*n* = 32,923) variations could be mapped on protein structures (*n* = 14,270). Step 2: Protein feature annotation. Forty protein features from seven main feature categories for the amino acid residues are collected from multiple databases, that is, DSSP (40) (version 3.0.2), PDBsum (41) (January 2018 update), PhosphoSitePlus (42) (February 2018 update), and UniProt (43) (release 2018_02). Step 3: Protein class annotation. The protein functional class annotations for genes are obtained from PANTHER (44) (release 13.1), Ensembl (version 93), and UniProt (43) (release 2018_02) databases. Step 4: Statistical analysis. Two-sided Fisher’s exact test is performed to identify the protein features that are significantly associated with pathogenic or population missense variations (after Bonferroni correction). The analysis is performed taking all variants in the DAGS1330 gene set, and then individually for groups of genes encoding proteins in 24 functional classes, to identify features of 3D mutational hotspots that are shared across all proteins as well as those that are unique to proteins performing a specific function.

To systematically identify the 3D features associated with “pathogenic” and “population” missense variants, we analyzed the 3D sites affected in 32,923 pathogenic (ClinVar and HGMD databases) and 164,915 general population variants (gnomAD database) from 1,330 disease-associated genes (Disease-Associated Genes with Structure [DAGS1330] set, Dataset S1; see *Materials and Methods* for details) using 14,270 experimentally solved human protein structures. We investigated a set of 40 3D features grouped in seven main feature categories reporting on the affected amino acid’s physicochemical properties (e.g., aromatic vs. charged or polar), structural context (e.g., α-helix, β-sheet, participation in hydrogen bonds), and their role in protein activity (i.e., “functional features,” such as their involvement in an enzyme’s active site, ligand binding pocket, cellular signaling, etc.). A brief outline of the study design and objectives is shown in Fig 1.

### Characteristic 3D Features of Pathogenic and Population Missense Variants of 1,330 Genes.

**Fig. 2.**
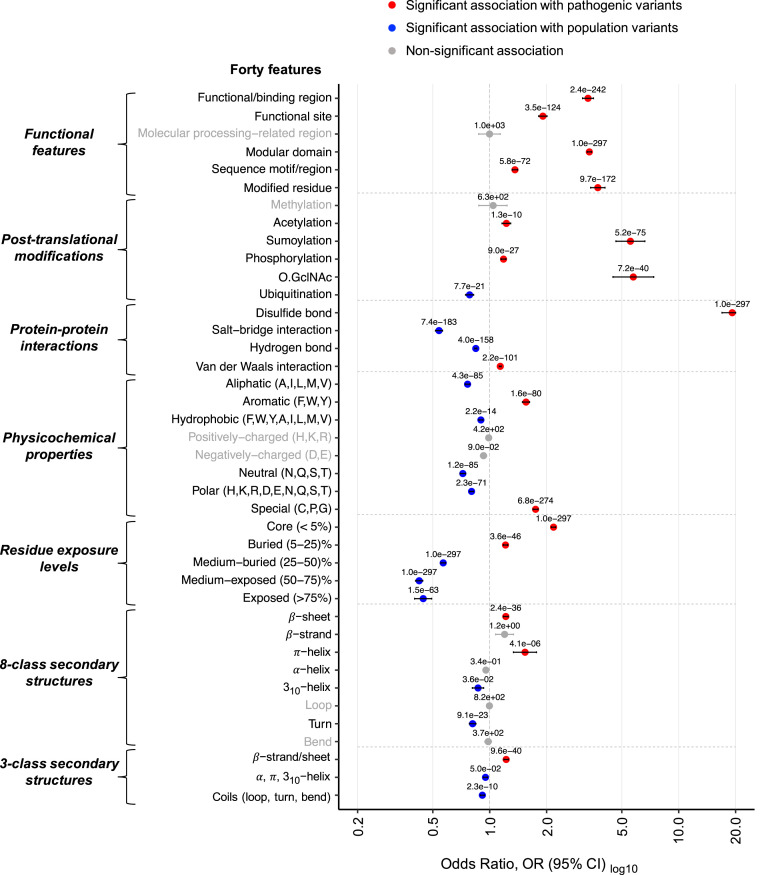
Association of pathogenic and population missense variations with 40 3D features (a combination of structural, physicochemical, and functional features of amino acids on protein structure) for 1,330 disease-associated genes (DAGS1330 set). The plot shows the results of two-sided Fisher’s exact tests of association between 32,923 pathogenic and 164,915 population amino acid variations with the features. Circles show the OR and are labeled with the q values (the corrected p values; see *Materials and Methods*), showing the significance of the association (a value of 1.0e-297 should be read as <1.0e-297, indicating the maximum significance), and the horizontal bars show the 95% CI. The OR > 1 and OR < 1, along with q < 0.05, indicate that the corresponding feature (*y* axis) is enriched in pathogenic (red circle) and population (blue circle) variants, respectively. The vertical dashed line at OR = 1 indicates no association between a variant type (pathogenic or population) and a feature. To facilitate the visualization, minimum and maximum values of OR along the *x* axis are set to 0.2 and 20.0, respectively. For nonsignificant association (q ≥ 0.05), the circle, CI bar, and feature names are gray.

By statistical association analysis of all variants from 1,330 genes together, we identified 18 out of 40 (45%) features that were significantly associated with pathogenic variants, while 14 out of 40 (35%) features showed significant association with population variants (Fig. 2). The remaining eight protein features (20%) showed no significant association with any variant type. In the rest of the paper, we will only report and discuss statistically significant results (with a corrected p value or “q” < 0.05; see *Materials and Methods* for details).

Disulfide bonds formed between covalently linked cysteines of two different proteins in a complex were found to have the highest enrichment of pathogenic missense variations among all investigated 3D features (19-fold; Fig. 2). The next highest burden for pathogenic variations was observed in the residues that were within 10 Å of posttranslational modification (PTM) sites in the structure [sites that undergo enzymatic addition of small molecules to certain amino acids after translation (45)], like SUMOylation (OR = 5.8) and O-linked *N*-acetylglucosamine (O.GlcNAc) (OR = 5.6; Fig. 2) sites. Instead, in population variants, the solvent-exposed residues in protein structures were observed to be the most affected 3D sites (OR = 0.4; Fig. 2).

Interestingly, the group of amino acids (Cys/C, Gly/G, and Pro/P) with peculiar characteristics (categorized as “special” in terms of their physicochemical properties in this study; Fig. 2), showed the highest association with pathogenic variations (twofold enrichment). Of these, cysteine (Cys) residues were found fourfold enriched in pathogenic variations (*SI Appendix*, Fig. S1*A*), consistent with the cogent association between variant pathogenicity and perturbation of disulfide bonds, as a Cys mutation will eliminate that bond. The three aromatic amino acids (Phe/F, Trp/W, Tyr/Y), both as a group (OR = 1.6; Fig. 2) and individually (*SI Appendix*, Fig. S1*A*), were found enriched for pathogenic mutations. Among them, tryptophan (Trp) residues, which are often involved in key molecular interactions (e.g., hydrophobic and cation–π interactions) (46) showed the strongest association (OR = 3.3; *SI Appendix*, Fig. S1*A*) with pathogenic variants.

Additionally, out of the six “functional features” indicating sites or regions of interest in proteins [as annotated in UniProt (43)], three were observed to be over threefold enriched in pathogenic variants, namely the “modular domain,” “modified residue,” and “functional/binding region” (Fig. 2). We also performed supplemental analyses on all of the 25 individual features that were initially collected from UniProt, then grouped (see *SI Appendix, Feature Set Mining and Annotation* for details), and analyzed as six categories in Fig. 2. Results of these fine-grained analyses revealed additional associations for pathogenic variants (output presented and discussed in full in *SI Appendix*, Fig. S1*B*).

### Characteristic 3D Features of Pathogenic and Population Missense Variants for Protein Functional Classes.

**Fig. 3.**
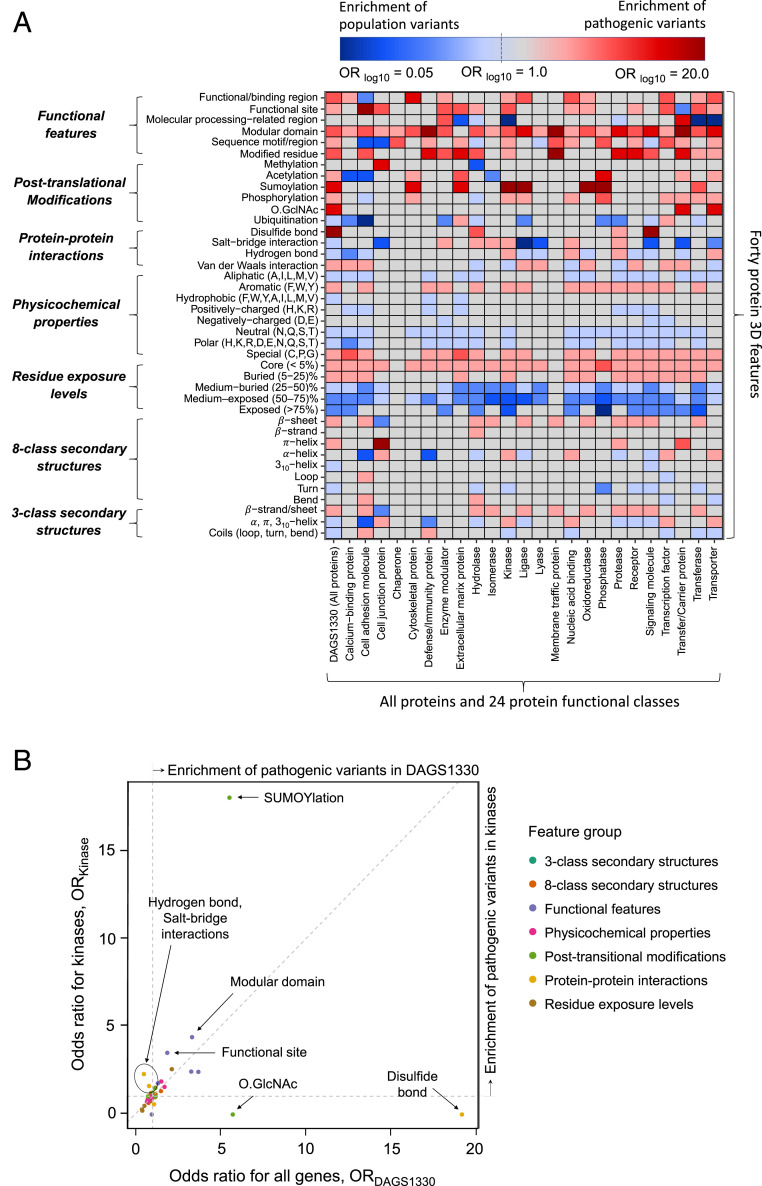
Some features of 3D mutational hotspots are conserved across different protein functional classes, whereas others are unique to specific classes. (*A*) Heatmap of ORs found from the burden analyses (two-sided Fisher’s exact test) on 40 3D features with pathogenic and population variants from all 1,330 disease-associated genes (full DAGS1330 dataset) and for subsets of genes grouped into 24 protein classes based on their molecular functions. To facilitate the visualization, minimum and maximum values of OR are set to 0.05 and 20.0, respectively. The red and the blue color gradients represent different degrees of association to pathogenic (1.0 < OR ≤ 20.0 and q < 0.05) and population (0.05 ≤ OR < 1.0 and q < 0.05) variants; darker color indicates stronger association. The gray cells in the heatmap represent features that are not significantly associated (q ≥ 0.05) with any variation type. Thus, the rows with only red or blue cells show the characteristic features of pathogenic or population variations that are consistent or conserved across all of the protein classes. In contrast, the rows with both red and blue cells indicate protein class-specific diverging features. (*B*) Scatter plot showing the correlation between the burden of pathogenic variations on different features for all genes along the *x* axis (${OR}_{DAGS1330}$) and for kinase protein class along the *y* axis (${OR}_{Kinase}$). Each circle represents a protein feature (indicated by an arrow), and has a different color according to the seven main feature categories. The diagonal line represents the agreement between the burden values found for all genes and those for kinases. The features above the diagonal line and to the left of the vertical line are enriched with pathogenic variations in kinases (hydrogen bond and salt bridge interaction sites), but are depleted of pathogenic variations in the full DAGS1330 set. The features above the diagonal line and to the right of the vertical line have an elevated burden of pathogenic variations in kinases (*y* axis), indicating that these features are more intolerant to substitutions for this protein class compared to the general trend for all proteins (*x* axis). In contrast, the features below the diagonal line and the horizontal line are enriched with pathogenic variations in the DAGS1330 set (disulfide bond and O.GlcNAc), but are depleted of pathogenic variations in kinases.

Protein structures present evidence for conserved regions that are relevant for a specific function, and such essential regions vary substantially in different protein functional classes (44, 47). We thus anticipate that features of 3D mutational hotspots in proteins performing different functions can differ from the general characteristics obtained by joint analysis of 1,330 genes. To identify such shared and/or unique function-specific 3D features, we quantified the burden of pathogenic variations compared to the population variations in 40 3D features separately for groups of genes encoding for similar protein functions (see Dataset S2 for protein class definition and annotated genes). The identified characteristic 3D features of pathogenic and population variants for all protein classes are listed in Dataset S3, and the output is summarized as a heatmap of enrichment values (odds ratio, OR) in Fig. 3*A*. The detailed Fisher’s exact test outputs for protein classes (OR, 95% CI and p values) are also presented in *SI Appendix*, Figs. S2#x2013;S8 for seven main feature categories.

Our class-specific analysis captured the 3D features that are susceptible to pathogenic mutations across all protein classes, indicating a crucial location for protein fitness. The residue exposure level (defined by the relative solvent accessible area [RSA] of that amino acid) is one such 3D feature type (Fig. 3*A*). Core (RSA < 5%) and buried (5% ≤ RSA < 25%) residues, which are usually embedded in a tight interaction network and are fundamental for protein stability, were found to be 3D hotspots of pathogenic mutations in all protein classes (Fig. 3*A* and *SI Appendix*, Fig. S4 *A* and *B*). Conversely, the residues relatively exposed to solvent (“medium-buried,” “medium-exposed,” and “exposed”; Fig. 3*A*) were found to be enriched with population variations in the majority of protein classes (*SI Appendix*, Fig. S4 *C*–*E*). Among the groups of amino acids, substitutions of aliphatic and neutral amino acids were found more likely to be tolerated in the general population (Fig. 3*A* and *SI Appendix*, Fig. S5 *B* and *G*) whereas mutations of aromatic and “special” amino acids were, on average, more pathogenic (Fig. 3*A* and *SI Appendix* Fig. S5 *C* and *H*) for all protein classes. Finally, the protein functional domain [according to the annotation available in UniProt (43)] was found to be a uniform hotspot of pathogenic mutations for all protein classes (Fig. 3*A* and *SI Appendix*, Fig. S8*D*).

Alongside the above described shared features, in numerous cases, we instead found marked differences between the 3D features of mutational hotspots of a specific protein class and those obtained from the joint analysis of the full DAGS1330 set (blue for those associated to population variants and red for pathogenic variants in Fig. 3*A*). For instance, from the all-gene analysis, we observed that pathogenic variations are more enriched in β-sheets (OR = 1.2, q = 2.4e-36) than in α-helices (OR = 0.9, q = 3.4e-01; Fig. 2), but the class-specific analysis highlighted significant enrichment of pathogenic mutations in α-helices for five protein classes (Fig. 3*A* and *SI Appendix* Fig. S3*D*): cell junction proteins (OR = 2.9), transcription factors (OR = 1.4), nucleic acid binding proteins (OR = 1.3), transporters (OR = 1.3), and kinases (OR = 1.2) (see *Discussion* for further details). A particularly informative example is that of kinases (Fig. 3*B*). Pathogenic variations for these enzymes were found to largely substitute the residues forming salt bridge interactions (${OR}_{Kinase}$ = 2.3 vs. ${OR}_{DAGS1330}$ = 0.5) and hydrogen bonds (${OR}_{Kinase}$ = 1.6 vs. ${OR}_{DAGS1330}$ = 0.8), whereas no association was observed with disulfide bonds, contrary to the trend found by the joint analysis on all genes (${OR}_{Kinase}$ = 0 vs. ${OR}_{DAGS1330}$ = 19.2; Fig. 3*B*). In addition to such a diverging pattern, for some features in kinases, we noticed an elevated burden of pathogenic mutations compared to the general trend for all proteins, indicating that these 3D sites are particularly important for the function of kinases. Examples of these features include SUMOYlation sites (${OR}_{Kinase}$ = 18.1 vs. ${OR}_{DAGS1330}$ = 5.6; Fig. 3*B*), modular domain residues (${OR}_{Kinase}$ = 4.4 vs. ${OR}_{DAGS1330}$ = 3.4; Fig. 3*B*), and functional sites (${OR}_{Kinase}$ = 3.5 vs. ${OR}_{DAGS1330}$ = 1.9; Fig. 3*B*). Similar results for other protein classes (*SI Appendix*, Table S1 and Figs. S2–S8) show that features of 3D mutational hotspots in proteins performing a specific function can substantially differ from the general trend, confirming the importance of our function-specific characterization of missense variants.

### Validation of 3D Features Associated with Pathogenic and Population Missense Variants on an Independent Set of Variants.

Having characterized the pathogenic and population missense variants using our set of 3D features (Figs. 2 and 3*A* and Dataset S3), we then carried out a comparison with an independent set of variants (see *Materials and Methods* for the preparation of the validation dataset) to assess how well we could recapitulate known pathogenic mutations and the potential of our identified features for helping with clinical interpretation of missense variants.

**Fig. 4.**
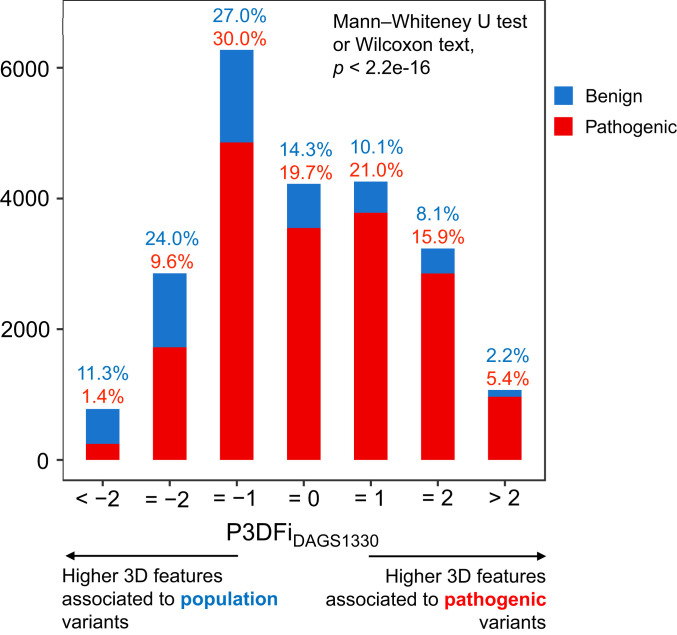
Distribution of pathogenic 3D feature index (P3DFi_DAGS1330_) values in an independent set of 22,695 variants (17,983 pathogenic and 4,712 benign). The plot shows the count of pathogenic and benign variants (*y* axis) in different P3DFi_DAGS1330_ bins (*x* axis) for 1,286 genes of all protein classes. The bin labels report the fraction of pathogenic and benign variants in each bin out of the total pathogenic and benign variants. In the plot, the pathogenic and benign variants show opposing distribution trends in the positive and negative P3DFi values (Mann–Whitney *U* test or Wilcoxon test of significance, p < 2.2e-06).

In order to quantify how deleterious an amino acid substitution is, we derived a pathogenic 3D feature index (P3DFi) per residue based on the difference between the pathogenic and population variant-associated 3D features of the reference (altered) amino acid (see details in *Materials and Methods*). We expect that the residues located in vulnerable 3D sites will have a higher number of pathogenic variant-associated features (P3DFi < 0). Conversely, residues substituted in benign variants are expected to have a greater number of population variant-associated features (P3DFi < 0). Thus, we calculated the P3DFi values for amino acids affected by 17,983 pathogenic and 4,712 benign missense variants of 1,286 genes. We then binned the variants based on their P3DFi values (from less than −2 to greater than 2), and, as expected, the pathogenic and benign variants showed opposite distributions (Mann–Whitney *U* test or Wilcoxon test of significance, p < 2.2e-06; Fig. 4) across different P3DFi_DAGS1330_ values, with P3DFi computed based on the 3D features associated with the pathogenic and population variants of all 1,330 genes. Note that the most positive (P3DFi > 2) and negative (P3DFi < −2) values represent the 3D sites with highest and lowest difference between pathogenic and population variant-associated 3D features identified in this study (Figs. 2 and 3*A*). Although a relatively small fraction of the total pathogenic and benign variants were in the highest and lowest index range, about 90% (967 out of 1,070) of all variants in the highest index range (P3DFi_DAGS1330_ > 2; Fig. 4) are pathogenic, and 68% (532 out of 779) of all variants in the lowest range (P3DFi_DAGS1330_ < −) are benign. We further compared this high-confidence classification of variants using P3DFi values with three state-of-the-art missense variant pathogenicity predictors, SIFT (11), PolyPhen2 (9), and CADD (48) (*SI Appendix*, Table S3). Both P3DFi_DAGS1330_ and P3DFi_Protein class_ (see *Materials and Methods* for details) performed comparably with these existing methods. Importantly, P3DFi_Protein class_ showed a better accuracy and precision than that of the P3DFi_DAGS1330_.

**Fig. 5.**
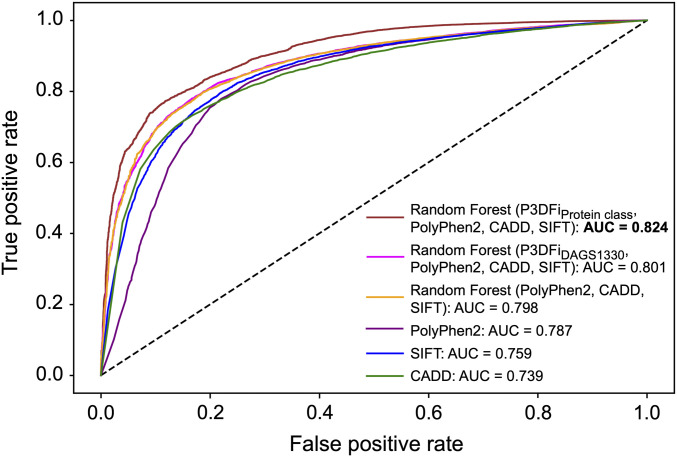
Comparison of the receiver operating characteristic (ROC) curves. The curves are drawn using the scores generated by six methods (Table 1) in predicting 22,362 variants (17,983 pathogenic and 4,655 benign). The plot is further labeled with the area under the curve (AUC) values. The “Random forest” ensemble model trained on P3DFi_Protein class_ (derived in this study), SIFT (11), PolyPhen2 (9), and CADD (48) scores provided the best AUC value of 0.824 (boldfaced).

However, it is worth noting that, unlike the other prediction scores, P3DFi values were not generated by a learning model that is trained on a set of features to classify pathogenic and benign variants. Instead, the P3DFi is purposely designed to characterize 3D mutational hotspots. We hypothesize that P3DFi values can serve as an orthogonal dimension in the variant pathogenicity prediction (*SI Appendix*, Fig. S9) with respect to the commonly used determinants employed by the existing methods. To test our hypothesis, we developed three ensemble models using the “Random forest” classifier (49) (see *Materials and Methods* for details): Two models were trained separately with the P3DFi_DAGS1330_ and P3DFi_Protein class_ values in addition to the scores from SIFT (11), PolyPhen2 (9), and CADD (48), and the third model was trained without any P3DFi values (Table 1). All three models were trained using the variants in the DAGS1330 dataset, and the performances were evaluated on the full validation dataset. The ensemble model including P3DFi_DAGS1330_ values in addition to the other prediction scores performed competitively with the one without any P3DFi and with the individual methods in terms of the balanced performance measured by the Matthews correlation coefficient (MCC) (Table 1), highlighting that P3DFi indeed carries valuable information for effectively determining variants’ pathogenicity. CADD score classified the variants with the highest true positive rate (∼90%), which, however, came at a cost of about 42% false positive rate. PolyPhen2, of the three independent predictors, showed the highest balanced accuracy (∼79%). For comparison, the ensemble model with P3DFi_Protein class_ scored the best balanced accuracy (∼82%), MCC (∼54%) (Table 1), and the highest area under the receiver operating characteristic (ROC) curve (82.4%) out of all six methods (Fig. 5), including the ensemble model with P3DFi_DAGS1330_.

**Table 1.** Comparison of the ensemble models using P3DFi (this work), SIFT (11), PolyPhen2 (HVAR) (9), and CADD (48) scores to the ensemble model without P3DFi values, and to the other individual scores

Method	Recall/ sensitivity/ true positive rate	Selectivity/ specificity/ true negative rate	Balanced accuracy	MCC	F1 score	Precision	Fallout/false positive rate	Miss rate/false negative rate
Random forest * (P3DFi_Protein class_, SIFT, PolyPhen2, CADD)	0.74	**0.91**	**0.82**	**0.54**	0.84	**0.97**	**0.09**	0.26
Random forest * (P3DFi_DAGS1330_, SIFT, PolyPhen2, CADD)	0.72	0.88	0.80	0.50	0.82	0.96	0.12	0.28
Random forest * (SIFT, PolyPhen2, CADD)	0.71	0.89	0.80	0.49	0.82	0.96	0.11	0.29
SIFT (11)	0.84	0.68	0.76	0.48	0.87	0.91	0.32	0.16
PolyPhen2 (9)	0.82	0.75	0.79	0.51	0.87	0.93	0.25	0.18
CADD (48)	**0.90**	0.58	0.74	0.48	**0.89**	0.89	0.42	**0.10**

The best score values are boldfaced. The performances are evaluated on 22,362 variants (17,707 pathogenic and 4,655 benign) from the validation set for which all of the scores were available. The training and test datasets are reported in Datasets S4 and S5, respectively, together with the scores used to develop all models and their outputs.

* Random forest ensemble model was developed using 2,000 decision tree classifiers (see details in Materials and Methods).

### Protein 3D Features Can Capture Missense Variations Leading to Protein Dysfunction.

**Fig. 6.**
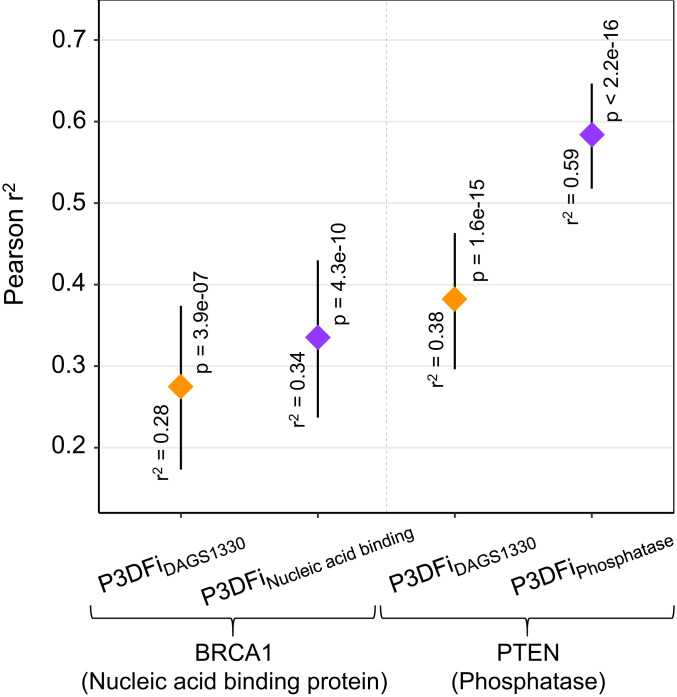
Comparison of the saturation mutagenesis screening readouts and P3DFi values (derived in this study). The figure shows the output of Pearson’s product moment correlation tests between the mean fitness scores from the mutagenesis experiment per amino acid (to all possible substitutions) and both the P3DFi_DAGS1330_ and P3DFi_Protein class_ values for two proteins: BRCA1 (50) and PTEN (51). The diamonds show the estimated correlation values (Pearson $r^{2}$). Vertical bars show the 95% CIs and are labeled with the significance (*p* values) of the test result. The correlation between experimental outputs measuring the functional consequence of mutations and the protein function-specific P3DFi (P3DFi_Phosphatase_ for PTEN and P3DFi_Nucleic acid binding_ for BRCA1) are higher than that of the P3DFi_DAGS1330_ values for both proteins. These results illustrate that 3D features specific to the protein function can provide a substantial advantage in correctly interpreting the consequences of missense variations.

Unlike in silico variant pathogenicity prediction scores, our analysis of 3D features can often provide additional and biologically interpretable information to rationalize the molecular effect of the variation on the protein. Here, we compare our feature-based characterization of 3D sites with the effect of amino acid substitutions obtained by functional assays for the enzyme PTEN and the DNA binding protein BRCA1 (Fig. 6).

We collected the saturation genome editing readouts for amino acid substitutions in the tumor suppressor gene *BRCA1* (13 exons in particular, encoding the RING and BRCT domains) from the literature (50). Subsequently, we quantified the P3DFi_DAGS1330_ and P3DFi_Nucleic acid binding_ for 326 residues in these two domains. While both the all-protein-based and function-specific P3DFi showed significant correlation (*p* = 3.9e-07 and 4.3e-10; Fig. 6) with the mutagenesis data, the P3DFi_Nucleic acid binding_ was 21% more correlated than the P3DFi_DAGS1330_. Notably, most of the residues with loss-of-function missense mutations (67%, 165 out of 248) annotated by the mutational screening also had a positive P3DFi_Nucleic acid binding_ value, agreeing with the P3DFi based classification of the consequence of the mutations (i.e., deleterious). Similarly, we collected the fitness scores for all PTEN residues derived using saturation mutagenesis experiments to annotate the loss-of-function and neutral missense variations (51). P3DFi_DAGS1330_ and P3DFi_Phosphatase_ were computed per amino acid: Again, P3DFi_Phosphatase_ showed a higher correlation ($r^{2}$ = 59%, *p* = 1.6e-15; Fig. 6) with the score quantifying the lipid phosphatase activity of PTEN than that of the P3DFi_DAGS1330_ ($r^{2}$ = 38%, *p* > 2.2e-16). In summary, more than 87% of the tested missense variations (768 out of 876) leading to reduced protein activity had positive P3DFi_Phosphatase_ values.

**Fig. 7.**
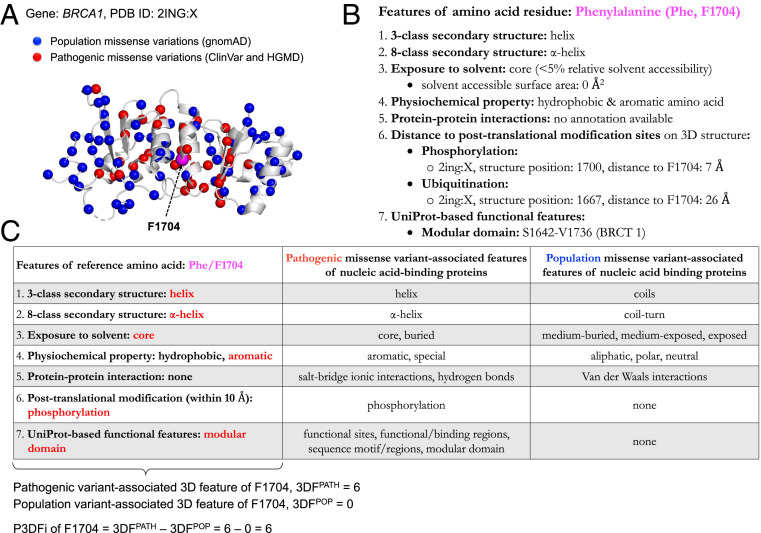
Protein features of missense variations on 3D structure provide intuitive insights into the effect of amino acid substitutions. (*A*) Structure (PDB ID code 2ING, chain: X) of BRCA1 with pathogenic (red) and population (blue) variations mapped, with an additional phenylalanine (Phe/F) at position 1704 (F1704) highlighted in pink for further analysis in this overview. (*B*) The 3D feature annotations for F1704. (*C*) Comparison of features of F1704 with protein class-specific 3D features associated to pathogenic and population variants (BRCA1 is annotated as a nucleic acid binding protein). A feature is highlighted in red if it matches a pathogenic variant-associated feature, or in blue if it matches a population variant-associated feature. In this example, F1704 possesses six pathogenic (${3DF}^{PATH}$) and zero population (${3DF}^{POP}$) variant-associated 3D features. Thus, for F1704, P3DFi_Nucleic acid binding_ is equal to 6 – 0 = 6 (a positive P3DFi value represents a 3D mutational hotspot).

Importantly, in addition to the quantitative index P3DFi, MIssense variant to protein StruCture Analysis web SuiTe [MISCAST (52); http://miscast.broadinstitute.org/] shows the structural, functional, and physicochemical properties of the mutated amino acid; these properties can help in generating intuitive hypotheses about the reason for protein dysfunction caused by the mutation, as illustrated by the following example. The phenylalanine (Phe/F) at position 1704 (F1704) of BRCA1 in Fig. 7*A* is highlighted as a magenta sphere along with the known pathogenic (ClinVar and HGMD) and population (gnomAD) variation positions rendered with red and blue spheres, respectively. Interestingly, ClinVar lists two missense variations of F1704 (F1704Y and F1704L), and both are of uncertain significance (VUS). The 3D features of F1704 (Fig. 7*B*) show that, besides being part of the BRCT (BRCA1 C-terminal) protein domain, the F1704 amino acid residue is located in the protein core with 0 Å^2^ RSA and in close proximity to a phosphorylation site (distance in sequence = 4 amino acids, distance in structure = 6.8 Å). Our results showed that residue exposure, substitution of aromatic residues, and close proximity of the altered 3D site to a phosphorylation site are features strongly associated with pathogenicity in nucleic acid binding proteins (Fig. 7*C*). Thus, mutation of F1704 could lead to structural defects by improper packing of the core and/or conformational changes near the phosphorylation site (Fig. 7*C*; positive P3DFi > 0 indicates a 3D mutational hotspot that is intolerant to substitution). Strikingly, all possible substitutions of Phe/F at position 1704 of BRCA1 have indeed been found to lead to loss of function in mutagenesis experiments (50), agreeing with our 3D features based output.

## Discussion

A large number of disease-associated missense variants is currently available in publicly accessible databases. However, the vast majority of them (about 75% of all clinically derived missense variants in ClinVar, October 2019 release) remain of uncertain significance (6), which is a major bottleneck in translational and clinical genetics.

An array of in silico tools are currently available that aid in the interpretation of sequence variants by providing a pathogenicity prediction score, generated using different classification algorithms, training cohorts, and features (30, 53). Some of the most commonly used tools are PolyPhen2 (9), SIFT (11), CADD (48), etc. While these variant pathogenicity prediction tools can classify pathogenic from benign variants with reasonable accuracy (65 to 80%) (54), they do not provide the user with the 3D structural context of the variation location, which could help explicate the functional impact of the variant. In fact, broader use of laboratory-based functional assays is still largely advocated for reliable inspection of the effect of missense variations on protein function (30, 55, 56). However, experimental approaches are not easily applicable or scalable, and require time, specialized skill sets, and equipment that are not widespread in the biomedical field.

In an effort to tackle this problem, and considering that the function of a protein is intimately linked to its structure, we hypothesized that characterizing the amino acid positions affected by pathogenic and benign missense variations in the context of the native protein structure can effectively decipher the molecular effects of a variant. Current studies analyzing missense variants in protein structures are mostly focused on the field of cancer research (22–24). Here we present a large-scale association study of germline pathogenic and population variations (*SI Appendix*, Fig. S10) with their respective 3D features for 1,330 genes, of which 1,077 are implicated in noncancer Mendelian disorders (4), using over 14,000 experimentally solved protein structures.

The only structure-related criterion currently recognized by the ACMG as a determinant of variant pathogenicity is the presence of missense variations in known functionally critical protein domains (30, 57). Our work also confirms the utility of these domains in variant pathogenicity assessment (over threefold enrichment of pathogenic variants in “modular domain”; Fig. 2). However, it is worth noting that 28% of the pathogenic missense variants in our dataset alter amino acids outside any domain currently annotated in UniProt (58), indicating that the complex interplay of factors governing protein stability and functionality cannot be reduced to domains alone. Indeed, we identified disulfide bonds and two PTM sites to have higher enrichment of pathogenic variations than that of “modular domains” (Fig. 2), in agreement with previous studies showing the link between pathogenic variants and these structure-based features (21) at a relatively small scale (i.e., variants from 1000 Genomes Project).

Several studies have discretely analyzed different structure-related features of amino acid positions (16, 59, 60) affected in missense variants. In contrast, we investigated a broad set of features, reporting on amino acids’ structural context as well as their chemical and functional properties. An example illustrating the benefit of our characterization using a more detailed set of features, for example, the eight-class description of secondary structures compared to the classical three classes (20, 61), is the identification of π-helical residues as a feature significantly associated with pathogenic variants (Fig. 2 and *SI Appendix*, Fig. S3*E*). Although relatively rare (about 0.6% of all residues in the 1,330 proteins analyzed in this study), 90 genes in our dataset had at least one disease-associated mutation changing a π-helical residue. These structural motifs are conserved and are known to contribute to the stabilization of specific binding sites within proteins (62, 63), so it is plausible that alteration of π-helices would be associated with serious perturbation of specific protein functions.

To the best of our knowledge, no previous studies have statistically assessed the properties of missense variants separately for genes encoding different protein functions, especially including structural information. Results obtained from analyzing variants from all 1,330 genes together revealed 3D features that are critical, in general, for protein fitness. These general characteristics of 3D hotspots that we (Fig. 2) and others (20–22, 64) have identified are necessary for making an educated guess about the effect of missense variations in any protein without knowing their function. But the insights gathered from these results are inherently limited because of the sheer diversity of proteins’ structural and functional properties. We thus delved deeper into the data and performed the same characterization of 3D mutational hotspots individually for 24 protein classes, which allowed us to identify many additional functionally relevant associations (Dataset S3 and Fig. 3*A*). Our results captured 3D features that have 1) a similar type and level of association (pathogenic or population, weak or strong) for every functional class (such as residues exposure level to solvent; Fig. 3*A*) and 2) an opposite or extremely elevated association for one/few classes compared to the general trend (i.e., function-specific characteristics). For example, results in Fig. 2 alone (from the all gene-based analysis) suggest that a variant where a 3D site involved in a hydrogen bond is mutated is likely a nonpathogenic variant. However, our function-specific analysis revealed four protein classes (transporter, protease, kinase, and nucleic acid binding proteins), for which residues forming interprotein hydrogen bonds are indeed associated with pathogenic variants (Fig. 3*A* and *SI Appendix*, Fig. S6*C*), similarly to what had been previously reported for a set of proteins and protein complexes (18). Conversely, amino acids near phosphorylation sites were found enriched for pathogenic variants in our joint analysis output (Fig. 3*A*, first column), recapitulating the findings of previous studies (59, 65, 66). But we observed an opposite pattern for hydrolases and cell adhesion molecules with frequent population variants near (<10 Å) phosphorylation sites (Fig. 3*A* and *SI Appendix*, Fig. S7*D*), further underlining the importance of our function-based analysis.

In addition to identifying function-specific features of missense variants in protein structures, our analysis could also explain contrasting claims found in the literature. In a recent review (20), both disease-causing and benign missense variations were reported to be predominantly located in helices and coil regions and less frequently in β-strands, whereas β-strands had been found to be more intolerant to mutations than α-helices in separate studies (61, 67). Our analysis of 1,330 genes did identify β-strands/sheets to be intolerant to substitution in general (Fig. 2), in agreement with the latter. Importantly, however, we also identified five protein classes (cell junction proteins, kinases, nucleic acid binding proteins, transcription factors, and transporters) that show enrichment of pathogenic variants in α-helical residues, in contrast to the general trend (Fig. 3*A* and *SI Appendix*, Fig. S3*D*). Interestingly, these residues tend to be relatively buried, with a probability of 46% (cell junction proteins) to 74% (kinases) of having a lower RSA than the average pathogenic variant-associated helical amino acid in the whole DAGS1330 set. These results show that characteristic features of 3D mutational hotspots vary based on the gene and variant set used for the analysis, which may be the reason underlying the diverging findings in the literature. Our study employing a unified dataset and workflow could detect such variability through the combined analysis over all genes and the individual analysis of specific functional protein classes (*SI Appendix*, Table S1 and Dataset S3). In particular, it is important to stress that we measured the statistical burden of pathogenic variations on a feature compared to the population variations with a two-sided Fisher’s exact test (see *Materials and Methods*). This method effectively reduces the possibility of obtaining a trivial result due to biased statistics of features in the proteins of a given functional class (e.g., finding α-helical residues significantly associated with pathogenic variants in predominantly α-helical proteins; *SI Appendix*, Fig. S11), and should therefore return only meaningful associations. For further verification of our protein class-specific results, we computed the “relative risk” (RR) (68) of a mutation to be pathogenic given that the altered residue has a 3D feature (for all 40 features) across the full dataset (DAGS1330 set) and for individual protein classes: Notably, the RR values were strongly correlated (Pearson $r^{2}$ = 94%) with the OR (Fig. 3), indicating that the ORs effectively approximate the RRs for our study.

The validity of this approach in yielding significant results is supported by the performance of a 3D feature-based index (P3DFi) that we generated in this study for each amino acid to quantify the relative effect of their substitution. For the same protein, P3DFi can be calculated considering the “general” characteristic 3D features of pathogenic and population variants found by analyzing all genes together (P3DFi_DAGS1330_) and also using the function-specific 3D features (P3DFi_Protein class_) (see *Materials and Methods*). Germline mutations in the phosphatase and tensin homolog (PTEN) protein have been shown to be associated with diverse clinical phenotypes, including cancers and autism spectrum disorder, due to the structural defects caused by the mutations (37, 51, 69). By comparing P3DFi values with the effect of *PTEN* variants on the protein’s lipid phosphatase activity in vivo as determined by saturation mutagenesis experiments (51), we noticed that the P3DFi_Phosphatase_ is 55% more correlated (Pearson $r^{2}$) with the mutational screen readouts than the P3DFi_DAGS1330_ (Fig. 6). To further validate our findings, we have also assessed the predictive value of P3DFi by evaluating the performance of a random forest classifier. When the same classifier is trained with P3DFi_Protein class_ in conjunction with other state-of-the-art variant pathogenicity prediction scores [from SIFT (11), PolyPhen2 (9), and CADD (48)], it can more accurately classify pathogenic and benign variants (8% higher MCC value; Table 1) than the model built using P3DFi_DAGS1330_ and the existing scores.

As is the case for functional classes, the characteristic features of 3D mutational hotspots can vary across different protein structural classes or folds (α–β barrel, β-propeller, α-horseshoe, etc.). Functional sites and regions have already been shown to be enriched with pathogenic variations (Fig. 2 and *SI Appendix*, Fig. S1*B* and refs. 64 and 70), and the 3D configuration of these sites/regions may well differ across different structural classes (71). Future investigations in this direction can unveil structure-specific insights into the impact of missense variations in different folds. It is important to note that we characterized the positions of missense variations on experimentally solved human protein structures as available in the PDB (8), which covers only one-third of the human proteome. For those cases where the gene is known but the corresponding protein structure has not yet been solved, inclusion of homology models could increase the power of statistical analyses similar to the one performed here. However, to ensure a reliable characterization of the 3D mutational hotspots, we employed only genes for which both pathogenic and population variations were mappable on experimentally solved structures, which still resulted in by far the largest study of this kind. It is also worth mentioning that, out of the total variants of the 1,330 disease-associated genes, we could map a higher proportion of pathogenic variants (61% of 63,606) onto protein structures compared to the population variants (33% of 496,869), which could plausibly be due to a bias of the relevant scientific community toward solving mainly the structure of the functionally relevant part of proteins (60) for structure-based target analysis and drug discovery purposes.

To summarize, in this study, we went beyond widely applied sequence- and conservation-based characterization of missense variants, and quantitatively determined the 3D protein features of amino acids affected by pathogenic or population variants from 1,330 disease-associated genes. Furthermore, we identified specific features that are important for the function of a certain protein class, adding one important dimension to our understanding of the functional effect of missense variations. We made the outcome of this study (precomputed P3DFi_DAGS1330_ and P3DFi_Protein class_ values for every possible amino acid exchange in proteins encoded by 1,330 disease-associated genes, along with the explicit listing of the 3D features of the altered site as the rationale for the index) available through a dedicated web server (MISCAST; http://miscast.broadinstitute.org/). By bringing the genetic variation into the 3D protein context, we believe that our study outcome can serve as a powerful resource for the translation of personal genomics to personal diagnostics and precision medicine: It can help to delineate variant pathogenicity, select candidate variants for functional assays, and aid in generating hypothesis for drug development.

## Materials and Methods

Detailed information is provided in *SI Appendix*.

### DAGS1330 and Validation Dataset Preparation.

Protein structures solved in human (in full or chimeric) were collected from the PDB (8). Protein-coding single nucleotide variants in the general population (hereafter referred to as “population” variant/variation) were obtained from gnomAD database, public release 2.0.2 (7). In addition, the “pathogenic” missense variations were collected from two sources: the ClinVar database (6), February 2018 release, and HGMD® professional release 2018.4 (5) (*SI Appendix*, Table S1). For 1,330 genes, we could map 164,915 population and 32,923 pathogenic variations onto 14,270 human protein structures (Fig. 1, step 1). This dataset is referred to as DAGS1330 (Dataset S1) and was used for the statistical analysis.

An additional validation set of pathogenic (*n* = 17,983) and benign (*n* = 4,712) missense variations was collected from ClinVar, February 2019 release, and HGMD® professional release 2019.2. All variants present in the DAGS1330 set were removed (*SI Appendix*). Further, high-throughput mutagenesis readouts, classifying loss-of-function variations from neutral ones in BRCA1 and PTEN, were collected from literature (50, 51).

### Protein Feature Mining and Annotation.

The amino acid residues were annotated with 40 protein features (a combination of structural, physicochemical, and functional features) from seven main feature categories (Fig. 1, step 2; see detailed definitions in *SI Appendix*). The secondary structure and solvent accessible area of amino acid residues were calculated using the DSSP (dictionary of protein secondary structure) program (40). Protein–protein interactions, PTMs, and functional features were obtained from the PDBsum (41), PhosphoSitePlus (42), and UniProt (43) databases, respectively.

### Protein Class Annotation.

The protein class information for the genes was aggregated from 1) PANTHER (Protein Analysis Through Evolutionary Relationships) database (44), 2) Ensemble family description (version 93), and 3) molecular function and/or biological process annotation available in UniProt (Fig. 1, step 3). Note that a protein may have multiple functions and so can be assigned into multiple classes (see the full list in Dataset S2).

### Statistical Analysis.

We used two-sided Fisher’s exact test of association to quantify the burden of pathogenic or population variations for each feature (Fig. 1, step 4). An estimate of enrichment or burden (OR), 95% CI, and the p value (p) showing the significance of the observed burden or association were obtained from the test output. All p values were corrected to generate “q” as p × 1,000 (total number of tests). Therefore, a 3D feature is considered to be a characteristic feature of pathogenic variants when the test outputs OR > 1 and q < 0.05. In contrast, when the test outputs OR < 1 and q < 0.05, the feature is referred to as a characteristic 3D feature of population variants.

### Computation of P3DFi per Amino Acid.

For each amino acid residue of the proteins encoded by the 1,330 disease-associated genes, we generated the 3D feature annotations (Fig. 7*B*) and counted the number of pathogenic and population variant-associated 3D features of the amino acid, denoted as ${3DF}^{PATH}$ and ${3DF}^{POP}$, respectively. Thereafter, the P3DFi per amino acid is computed as ${3DF}^{PATH}$ minus ${3DF}^{POP}$ (P3DFi 0 thus indicates a 3D mutational hotspot; Fig. 7*C*). Note that we identified the pathogenic and population variant-associated 3D features for all 1,330 genes analyzed together as one pool (Fig. 2) and also for 24 different protein classes (Fig. 3*A*). Therefore, P3DFi can be derived using the full DAGS1330-based 3D features (P3DFi_DAGS1330_) and also using protein class-specific (P3DFi_Protein class_) 3D features.

### Development of Ensemble Model.

All models were developed using the classical random forest method, which was implemented using the scikit-learn machine learning library for Python. The model parameters were set to number of estimators or decision trees = 2,000, quality measure = “gini,” and the maximum depth of the trees = 10. Both the training and test datasets, along with the prediction scores (>0.5: deleterious/pathogenic, ≤0.5: neutral/benign), are available in Datasets S4 and S5.

## Supplementary Material

Supplementary File

Supplementary File

Supplementary File

Supplementary File

Supplementary File

Supplementary File

## Data Availability

All data that are used and generated in this study are made available through Datasets S1–S5 and the MISCAST webserver (http://miscast.broadinstitute.org/).
