# Supplementary material for: Comprehensive characterization of amino acid positions in protein structures reveals molecular effect of missense variants
Source: Proc Natl Acad Sci U S A. 2020 Oct 26;117(45):28201–11. doi: 10.1073/pnas.2002660117 (PMC7668189; doi:10.1073/pnas.2002660117)
Supplement: Supplementary File [file pnas.2002660117.sapp.pdf]

PNAS
www.pnas.org

Supplementary Information for

Comprehensive characterization of amino acid positions in protein structures reveals molecular effect of missense variants

Sumaiya Iqbal, Eduardo Pérez-Palma, Jakob B. Jespersen, Patrick May, David Hoksza, Henrike O. Heyne, Shehab S. Ahmed, Zaara T. Rifat, M. Sohel Rahman, Kasper Lage, Aarno Palotie, Jeffrey R. Cottrell, Florence F. Wagner, Mark J. Daly, Arthur J. Campbell and Dennis Lal

Sumaiya Iqbal, Arthur J. Campbell, and Dennis Lal.
E-mail: sumaiya@broadinstitute.org, arthurc@broadinstitute.org, and lald@ccf.org

This PDF file includes:

Supplementary text
Figs. S1 to S11
Tables S1 to S3
Legends for Dataset S1 to S5
SI References

Other supplementary materials for this manuscript include the following:

Datasets S1 to S5

www.pnas.org/cgi/doi/10.1073/pnas.2002660117

Supporting Information Text

Disease-Associated Genes with Structure (DAGS1330) and Variant Set Preparation. The Protein Data Bank (PDB) (1) was searched in January 2018 for all available human (in full or chimeric) protein structures, resulting in 43,805 experimentally solved structures. From UniProt (2) release 2018_02, 5,870 unique protein identifiers and $5,850$ unique gene names for these 43,805 structures were collected. Mapping of coordinates of amino acid residues in the 3D structure to linear protein sequence was derived from the SIFTS database (3).

Protein-coding single nucleotide variants (SNVs) in the general population were retrieved from gnomAD, public release 2.0.2 (4), which we refer to as population variant/variation in this study. Missense variant annotations were extracted from gnomAD Variant Call Format (VCFs) (http://gnomad.broadinstitute.org/downloads) file using vcftools (5). Entries from the canonical transcript (CSQ canonical = "YES" flag), passing gnomAD standard quality controls (Filter = "PASS" flag) were extracted. The canonical transcript is defined as the longest CCDS translation with no stop codons according to Ensembl (6).

We collected patient missense variant data from two sources: the ClinVar database (7), February 2018 release and the Human Gene Mutation Database (HGMD®) Professional release 2017.2 (8). ClinVar variants were downloaded directly from the ftp site (ftp://ftp.ncbi.nlm.nih.gov/pub/clinvar/) in table format and were subsequently filtered to keep missense variants with "Pathogenic" and/or "Likely Pathogenic" clinical consequence. Raw HGMD files were filtered to collect missense mutations with high confidence (confidence = "HIGH" flag) and disease-causing state (variantType = "DM" flag, indicating Disease Mutation). All annotations refer to the human reference genome version GRCh37.p13/hg19. The combined set of variants from ClinVar and HGMD databases is hereafter referred to as pathogenic variants and the corresponding single amino-acid substitutions as pathogenic variations.

Altogether, we collected $1,485,579$, $16,570$, and $47,036$ amino acid-altering missense variants from gnomAD, ClinVar and HGMD databases, respectively. These variants were from $5,850$ human genes encoding proteins with $43,805$ structures available in the Protein Data Bank (PDB) (1). We filtered out genes for which (*i*) canonical isoform protein sequences were not translated from canonical transcripts and (*ii*) no altered amino acid residue was mappable to a protein structure (*SI Appendix*, Table S1). The resulting dataset included $1,330$ genes, for which there was at least one pathogenic and population missense variation that we could map on one or more protein structure. Therefore, we included only these $1,330$ genes with $164,915$ population and $32,923$ pathogenic variations mappable on $14,270$ human protein structures in our final dataset, referred to as Disease-Associated Genes with Structure (DAGS1330) dataset in this study. The list of genes in DAGS1330 is available in Dataset S1. The missense variations in the DAGS1330 set were used to perform the statistical analyses and the outcome (characteristic features of pathogenic and population missense variation position in 3D) was validated on independent test sets.

Validation Dataset Preparation. A validation set of pathogenic and benign missense variant was collected from the ClinVar (February 2019 release) and HGMD® (professional release 2019.2) databases. All variants present in the DAGS1330 set were removed. The remaining set included $4,712$ ClinVar benign and likely benign variants (hereafter collectively referred to as benign) and $17,983$ pathogenic variants (ClinVar pathogenic and likely pathogenic variants and HGMD-reported disease mutations). The validation set contained benign and pathogenic variants from 1,286 genes out of the $1,330$ genes in the DAGS1330 set. Further, high-throughput mutagenesis readouts, classifying loss-of-function or damaging variations from neutral ones in BRCA1 and PTEN were collected from literature (9, 10)

Feature set mining and annotation. We annotated the amino acid residues positions in proteins encoded by $1,330$ genes with forty (a combination of structural, physicochemical and functional) features from seven main groups. In the following, we introduce the features, and describe their curation and annotation process.

1. Three-class secondary structures (feature count: 3). Protein secondary structure (SS) is the three-dimensional (3D) form of local segments of proteins that are defined by the hydrogen bonds between the side chain atoms of amino acids in the peptide backbone. We obtained precomputed SS assignment values for the protein structures from the DSSP repository (11). Based on the hydrogen bond energy, eight different types (see below) of SS were assigned to the amino acid residues in the protein structure. These eight different types of SSs can be grouped into three broader categories: (*i*) β-sheet/strand: β-strand and extended β-sheet; (*ii*) Helices – 3_{10}-helix, α-helix, π-helix; (*iii*) Coils – turn, bend, and random loops. For proteins with multiple available structures, we derived a consensus annotation which represents the maximum agreement of SS type assignments out of all structures.

2. Eight-class secondary structures (feature count: 8). We have also looked into more detailed types of SSs to check for associations with pathogenic or population variants. The eight structure types collected from DSSP are: (*i*) β-strand – residues in isolated β-bridge; (*ii*) β-sheet – residues in extended strand that participates in β-ladder; (*iii*) 3_{10}-helix – hydrogen bond between i^{th} and $(i+3)^{th}$ residues to build each helical turn; (*iv*) α-helix – hydrogen bond between i^{th} and $(i+4)^{th}$ residues to build each helical turn; (*v*) π-helix – hydrogen bond between i^{th} and $(i+5)^{th}$ residues to build each helical turn; (*vi*) turn – hydrogen bonded turn; (*vii*) bend – residues of high curvature where the angle between C_iC_{i+2} and $C_{i-2}C_i$ is at least 70°; (*viii*) loop – random loop where no other rule applies. Similar to the three-class SS annotation, for proteins with multiple structures, we considered the maximum agreement of SS type assignments from all the available structures as the final annotation.

3. Residue exposure level (feature count: 5). The solvent accessible surface area (ASA) of each amino acid residue in the protein structures was also collected from DSSP repository (11). The ASA is measured in units of square of Angstroms

(Å^2). The Relative solvent accessible area (RSA) of a residue "X" was calculated by normalizing the ASA of that residue by the ASA of the same residue in a reference tripeptide state (Gly-X-Gly), collected from (12). When multiple structures were available for an amino acid, we considered the median of all RSA values for that residues. Based on the value of RSA, we labeled each amino acid with one of the following five types: (*i*) core (RSA $< 5\%$); (*ii*) buried ($5\% \leq$ RSA $< 25\%$); (*iii*) medium-buried ($25\% \leq$ RSA $< 50\%$); (*iv*) medium-exposed ($50\% \leq$ RSA $< 75\%$); (*v*) exposed (RSA $\geq 75\%$).

4. Physicochemical properties of amino acids (feature count: 8). While every amino acid have an amino and a carboxyl group, their side chains are all different and have distinct physicochemical properties that influence protein conformation and function. Based on the property of the side chain, the 20 natural amino acids are divided into eight groups: (*i*) aliphatic – alanine (Ala/A), isoleucine (Ile/I), leucine (Leu/L), methionine (Met/M), valine (Val/V); (*ii*) aromatic – phenylalanine (Phe/F), tryptophan (Trp/W), tyrosine (Tyr/Y); (*iii*) hydrophobic – aliphatic and aromatic amino acids; (*iv*) positively-charged – arginine (Arg/R), histidine (His/H), lysine (Lys/K); (*v*) negatively-charged – aspartic acid (Asp/D), glutamic acid (Glu/E); (*vi*) neutral – asparagine (Asn/N), glutamine (Gln/Q), serine (Ser/S), threonine (Thr/T); (*vii*) polar – positively-charged, negatively-charged, and neutral amino acids; (*viii*) "special" – proline (Pro/P): has a cyclic side chain and cannot make backbone hydrogen bonds; glycine (Gly/G): does not have a side chain and allows flexibility, cysteine (Cys/C): the side chain has a reactive sulfhydryl group. In our analysis, we considered only the amino acid residues that have defined coordinates in protein 3D structures.

5. Protein-protein interactions (feature count: 4). The PDBsum database (13) was curated to annotate amino acid residues with residue-residue interaction types between amino acid pairs at distance ≤ 4 Å on the protein-protein interface of complex structures. An amino acid can possibly be involved in different bonds in multiple protein complexes. Here, we kept records of all the interactions that one residue was involved in different conformations. The four types of interactions/bonds that we considered are: (*i*) disulfide bond – a covalent bond between cysteine side chains, yielding a disulfide bridge, which is an key determinant of protein 3D structure; (*ii*) salt-bridge interaction – an ionic bond between oppositely charged residues; (*iii*) hydrogen bond – electrostatic interaction between two atoms bearing partial negative charges, that share a partially positively charged hydrogen; (*iv*) Van der Waals interaction – a bond formed by the transient and weak Van der Waals attraction between two close atoms.

6. Post-translational modifications (feature count: 6). Post-translational modification (PTM) refers to the covalent and enzymatically-mediated modification of proteins to form the mature protein. We collected amino acid positions of six different PTM types from the PhosphoSitePlus database (14): (*i*) acetylation – introduces an acetyl group; (*ii*) methylation – addition of a methyl group; (*iii*) O.GlcNAc – also known as O-linked N-acetylglucosamine, is a form of protein glycosylation; (*iv*) phosphorylation – attachment of a phosphoryl group; (*v*) SUMOylation – addition of SUMO (small ubiquitin-like modifiers) molecule; (*vi*) ubiquitination – attachment of ubiquitin. Upon mapping the PTM types onto the protein structure, we computed the spatial distances between the C_α atoms of each amino acid and different PTM sites in the structure. We annotated each amino acid with the nearest distance to the six PTM types, where available. For our analysis, we considered the amino acids that are spatially close to a PTM site, i.e. if the residue is located within < 10 Å distance from the PTM site in the structure.

7. Functional features (feature count: 6). UniProt database (2) was mined to collect the sequence annotations, describing regions or sites of interest in protein in terms of 25 features. Genomic annotations were download as bed files from the UniProt webpage (ftp://ftp.uniprot.org/pub/databases/uniprot/current_release/knowledgebase). All hg38 coordinates were converted to hg19 coordinates using the UCSC liftover tool (https://genome.ucsc.edu/cgi-bin/hgLiftOver). Only correctly lifted-over regions were then reformatted to ANNOVAR (15) region format files for hg19 (http://annovar.openbioinformatics.org/en/latest/user-guide/input/). Pathogenic and population VCF files were annotated using ANNOVAR with the different UniProt features. Guided by EMBL-EBI online material (https://www.ebi.ac.uk/training/online/course/UniProt-exploring-protein-sequence-and-functional-information/sequence-features) and similarity, these 25 features were coarse grained into six categories to increase the power of the statistical association tests performed in this study. These six feature categories are (*i*) functional site – active site, metal binding site, binding site, site; (*ii*) functional/binding region – zinc finger, DNA binding region, nucleotide phosphate binding region, calcium binding region; (*iii*) sequence motif/region – region, repeat, coiled coil, motif; (*iv*) modular domain – domain, topological domain, transmembrane, intramembrane; (*v*) molecular processing-related region – peptide, transit peptide, signal peptide, propeptide; (*vi*) modified residue – modified residue, lipidation, disulfide bond, cross-link, glycosylation. If an amino acid residue is part of any subtype of a category, that residue is annotated by the corresponding category.

Protein Class Annotation. To identify the properties of pathogenic and population missense variations on structure at different protein function level, we grouped the 1,330 genes into twenty-four major protein classes (main text, Fig. 1, Step 3) using the PANTHER database (Protein ANalysis THrough Evolutionary Relationships (16), 13.1 release, published in February 2018). After automatic annotation, 624 genes were not assigned to any specific protein class. We then collected the protein class information for these 624 genes from (*i*) the Ensemble family description (version 93) using BioMart (17) and (*ii*) molecular function and/or biological process annotation available in UniProt (release 2018_02) as defined by the Gene Ontology consortium (18). The list of genes, number of protein 3D structures and missense variations mapped on structures for the protein classes are given in Dataset S2. Note that a protein may have multiple functions and so can be assigned into multiple protein classes.

Statistical Analysis. The two-sided Fisher's exact test of association was performed for each of the forty 3D features, taking the counts of pathogenic and population missense variations with and without a feature, to quantify the burden of pathogenic or population variations for each feature (main text, Fig. 1, Step 4). An estimate of enrichment or burden (odds ratio, OR), 95% confidence interval (CI) of the OR value, and the p-value (p) showing the significance of the observed burden or association, were obtained from the test output. The described analysis was performed on the full DAGS1330 set and separately on subsets of variations in genes grouped in twenty-four protein functional classes. Thus, we performed a total of 40 (number of features) $\times$ 25 (number of datasets) = 1000 tests. Subsequently, the p-values were corrected to generate a corrected value of p that we called "q", calculated as $p \times 1000$ according to the Bonferroni correction for multiple testing in statistical analysis. Therefore, a 3D feature is considered to be a characteristic feature of pathogenic variants when the test outputs OR > 1 and $q < 0.05$. In contrast, when the test outputs OR < 1 and $q < 0.05$, the feature is referred to as a characteristic 3D feature of population variants. This approach for the characterization of variants by comparative enrichment analysis taking both pathogenic and population variants into account in the two-sided Fisher's exact test, reduces the possibility of obtaining a result simply because of the abundance of a certain 3D feature in a protein class. For example, the fraction of residues located in the protein core ("core" residues, see "Residue exposure level" ranking in *Materials and Methods*) that are mutated in both pathogenic and population variants (39% vs. 23%) were used to derive the enrichment of pathogenic variations in the protein core (>2-fold, Fig. 2). Thanks to our comparative method we can be reasonably confident that if we see an enrichment of pathogenic variations in protein core positions, it is not only because our sample contains a high proportion of compact structures where the majority of residues are buried.

For further verification of our protein class-specific results by OR enrichment analysis (main text, Fig. 3A), we computed the "relative risk (RR)" (19) of a mutation to be pathogenic given the altered residue has a 3D feature (for all forty features) across the full dataset (DAGS1330 set) and for individual protein classes: Notably, the RR values were strongly correlated (Pearson $r^2 = 94\%$) with the odds ratios (ORs, main text, Fig.3A), indicating that the ORs effectively approximate the RRs for our study (20, 21).

Computation of Pathogenic 3D Feature Index (P3DFi) per Amino Acid. For each amino acid residue of the proteins encoded by the $1,330$ disease-associated genes, we generated the 3D feature annotations (available in MISCAST) and counted the number of pathogenic and population variant-associated 3D features of the amino acid, denoted as $3DF^{PATH}$ and $3DF^{POP}$, respectively. Thereafter, the pathogenic 3D feature index (P3DFi) per amino acid is computed as $3DF^{PATH}$ minus $3DF^{POP}$ (P3DFi > 0 thus indicates a 3D mutational hotspot). Note that we identified the pathogenic and population variant-associated 3D features both for all $1,330$ genes analysed together as one pool (main text, Fig. 2) and also for twenty-four different protein classes, grouping genes encoding for similar functions (main text, Fig. 3A). Therefore, P3DFi can be derived using the full DAGS1330-based 3D features ($P3DFi_{DAGS1330}$) and also using protein class-specific ($P3DFi_{Protein\ class}$) 3D features. When a protein belongs to more than one protein class, a single $P3DFi_{Protein\ class}$ per amino acid was decided by taking the majority agreement from all $P3DFi_{Protein\ class}$ and $P3DFi_{DAGS1330}$ values. The $P3DFi_{DAGS1330}$ and all $P3DFi_{Protein\ class}$ values per amino acid of the 1,330 proteins are made available through the MISCAST web server (http://miscast.broadinstitute.org/)

Development of Ensemble Model. Annotations of variants in the DAGS1330 set (used as the training set to develop the model) and the validation set with predicted pathogenicity scores from SIFT (22) (SIFT_score), PolyPhen2 (23) (Polyphen2_HVAR_score) and CADD (24) (CADD_phred) were generated using ANNOVAR (15). The categorical prediction (1: deleterious/pathogenic, -1: neutral/benign) was determined using the guidelines described in the Annovar documentation (15). Two ensemble models were developed using the scores from the three existing methods, and separately with $P3DFi_{DAGS1330}$ and $P3DFi_{Protein\ class}$ values. An additional ensemble model was trained only with the scores from existing methods (i.e. without any P3DFi). All the models were developed using the classical random forest method using: Number of estimators or decision tree classifiers = $2,000$, quality measure as "gini", and the maximum depth of the trees = 10. To eliminate the imbalanced proportion of pathogenic and benign variants from the training dataset (ratio of pathogenic to benign variants > 5), and ensure robust training of the models, a random oversampling of pathogenic variants was performed. The numerical prediction outputs generated by the models were categorized by applying the default threshold of 0.5 (>0.5: deleterious/pathogenic, $\leq$0.5: neutral/benign). Both the training and test datasets, along with the predictions generated by the ensemble models, are available in Dataset S4 and S5.

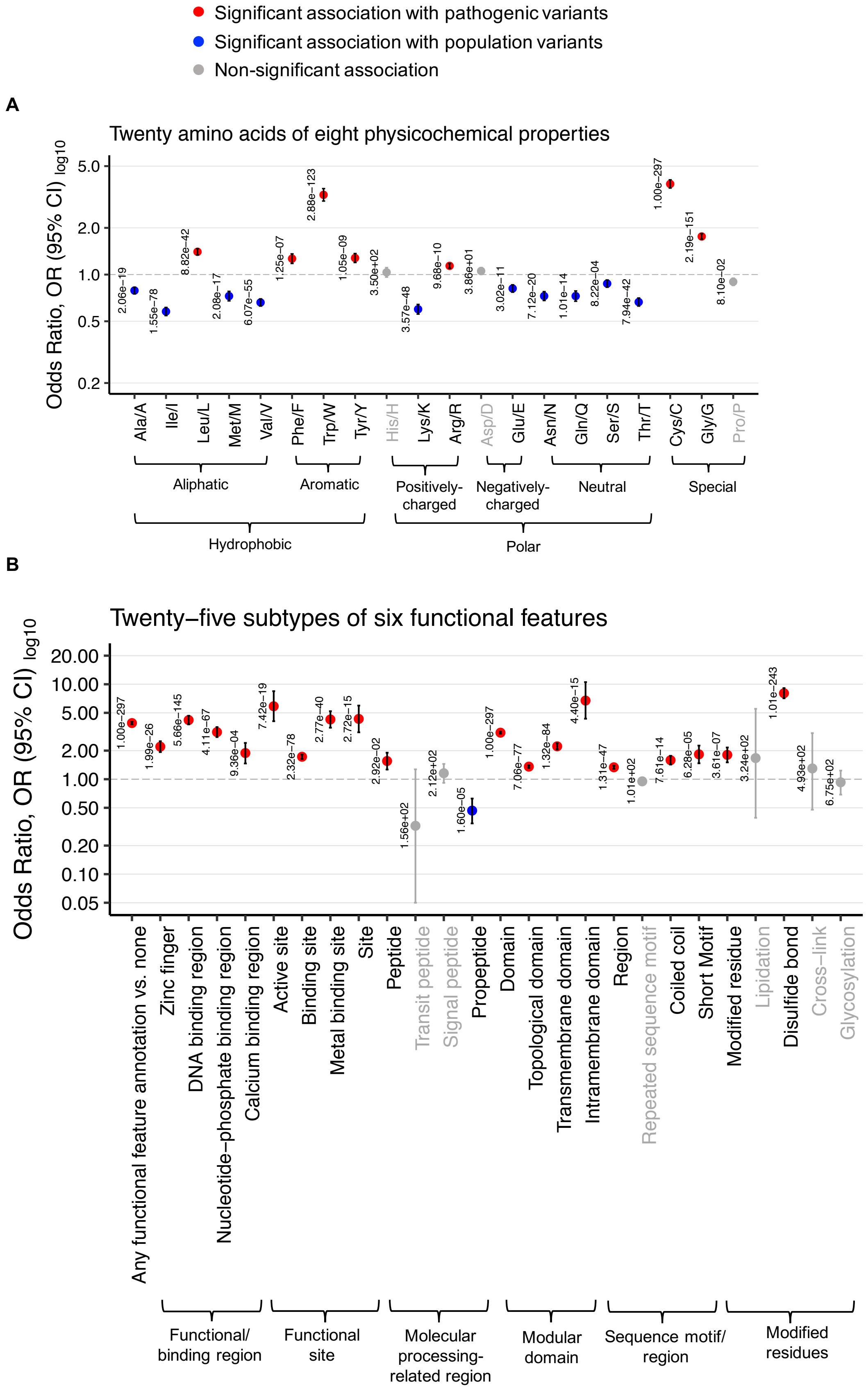
Significant association with pathogenic variants
Significant association with population variants
Non-significant association
A
Twenty amino acids of eight physicochemical properties
Odds Ratio, OR (95% CI) log10
Ala/A
Ile/I
Leu/L
Met/M
Val/V
Phe/F
Trp/W
Tyr/Y
His/H
Lys/K
Arg/R
Asp/D
Glu/E
Asn/N
Gln/Q
Ser/S
Thr/T
Cys/C
Gly/G
Pro/P
Aliphatic
Aromatic
Positively-charged
Negatively-charged
Neutral
Special
Hydrophobic
Polar
B
Twenty–five subtypes of six functional features
Odds Ratio, OR (95% CI) log10
Any functional feature annotation vs. none
Zinc finger
DNA binding region
Nucleotide–phosphate binding region
Calcium binding region
Active site
Binding site
Metal binding site
Site
Peptide
Transit peptide
Signal peptide
Propeptide
Domain
Topological domain
Transmembrane domain
Intramembrane domain
Region
Repeated sequence motif
Coiled coil
Short Motif
Modified residue
Lipidation
Disulfide bond
Cross–link
Glycosylation
Functional/ binding region
Functional site
Molecular processing-related region
Modular domain
Sequence motif/ region
Modified residues

Fig. S1

Fig. S1. (Previous page.) Association of pathogenic and population missense variants from 1,330 disease-associated genes (DAGS1330 set) with (*A*) twenty natural amino acids (grouped into eight physicochemical properties and analyzed in the main text, **Fig. 2**), (*B*) 3D sites with twenty-five functional features collected from UniProt (coarse grained into six features and analyzed in the main text, **Fig. 2**). The plots show the results of two-sided Fisher's exact tests of association between 32,923 pathogenic and 164,915 population amino acid variations with the features. Circles show the odds ratio (OR) and are labelled with the corrected p or "q" values, showing the significance of the association (a value of 1.0e-297 is to be read as <1.0e-297, indicating maximum significance), and the horizontal bars show the 95% confidence interval (CI). The OR $>$ 1 and OR $<$ 1, along with $q <$ 0.05, indicate that the corresponding feature (y-axis) has an enrichment of pathogenic (red circle) and population (blue circle) variants, respectively. The OR = 1 (dashed vertical line) indicates that there is no association between a variant type (pathogenic or population) and a feature. To facilitate the visualization, minimum and maximum values of OR along x-axis are set to 0.05 and 20.0, respectively. For non-significant association (q $\geq$ 0.05), the circle, CI bar and feature names are grey. Out of the three amino acids that are categorized to have "special" physicochemical properties in this study, Cys and Gly had significant enrichment of pathogenic variations (in *A*). The Cys enrichment is linked to the strong association between disulfide bond and pathogenic variants (main text, **Fig. 2**). Glycine is the smallest amino acid and is predominantly found in flexible parts of the protein structure (more than 67% of the Gly reference amino acid residues in our dataset form pliable coils). Association between the substitution of Gly amino acid and pathogenic variants (2-fold) can be due to the steric clashes introduced by the substitution, causing destabilization of the protein structure. While all the aromatic amino acids were found enriched for pathogenic mutations, all the aliphatic amino acids, only except Leu, were found tolerant to substitution. Out of the positively-charged amino acids, Arginine (Arg/R, OR = 1.1, $q <$ 9.7e-10) alone had a modest but significant burden of pathogenic mutations (in *A*). The supplemental analysis of the twenty-five individual features collected from UniProt (in *B*) allowed us to quantify the burden of pathogenic variants for non-membrane topological domains (OR = 1.4, q = 7.1e-77), transmembrane (OR = 2.2, q = 1.3e-84), and intramembrane domains (OR = 6.7, q = 4.4e-15) of membrane-spanning proteins. In addition to recapitulating features like short biologically-relevant motifs, DNA binding sites, and zinc fingers, that were known to be frequently impaired in pathogenic variants (25, 26), our study revealed novel associations as well. Some notable examples of features that were not previously linked with the germline, primarily non-cancer (91% in our dataset, **Fig. S10**) pathogenic variants are active sites (OR = 5.9, q = 7.4e-19), metal-binding sites (OR = 4.3, q = 2.8e-40), coiled-coils (OR = 1.6, q = 7.6e-14) and short, biologically-active peptides (OR = 1.6, q = 2.9e-02), highlighting the importance of these 3D sites for the function of a protein and their putative intolerance to perturbation by missense variation.

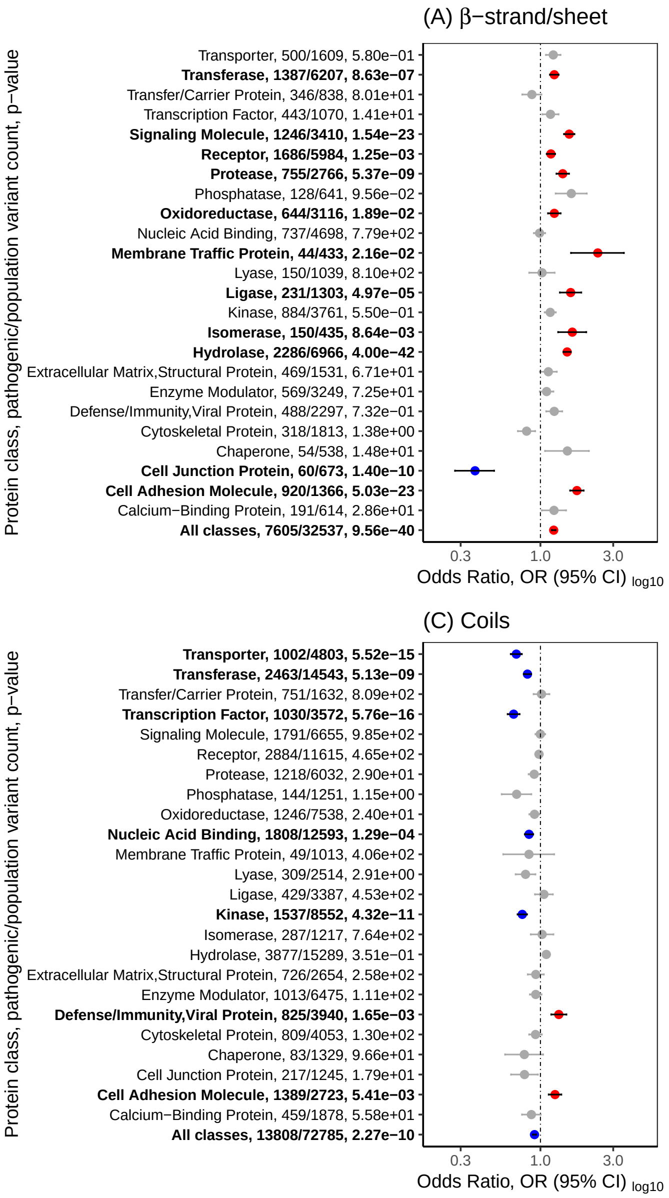

Fig. S2. Enrichment of pathogenic and population missense variants for "All classes" and twenty-four protein functional-classes in the three-class secondary structure types. The plot shows the results of two-sided Fisher's exact tests of association. Circles show the odds ratio (OR) and the horizontal bars show the 95% confidence interval (CI). The y-axis is labelled with the protein class name, counts of pathogenic and population variants with the corresponding feature, and corrected p or "q" values, showing the significance of the association (a value of 1.0e-297 should be read as <1.0e-297, indicating maximum significance). The OR $>$ 1 and OR $<$ 1, along with $q <$ 0.05, indicate that the corresponding feature (plot label) has an enrichment of pathogenic (red circle) and population (blue circle) variants, for the respective protein class (y-axis). The OR = 1 (dashed vertical line) indicates that there is no association between a variant type (pathogenic or population) and a feature. To facilitate the visualization, minimum and maximum values of OR along x-axis are set to 0.05 and 20.0, respectively. For non-significant association ($q \geq$ 0.05), the circle and CI bar are grey.

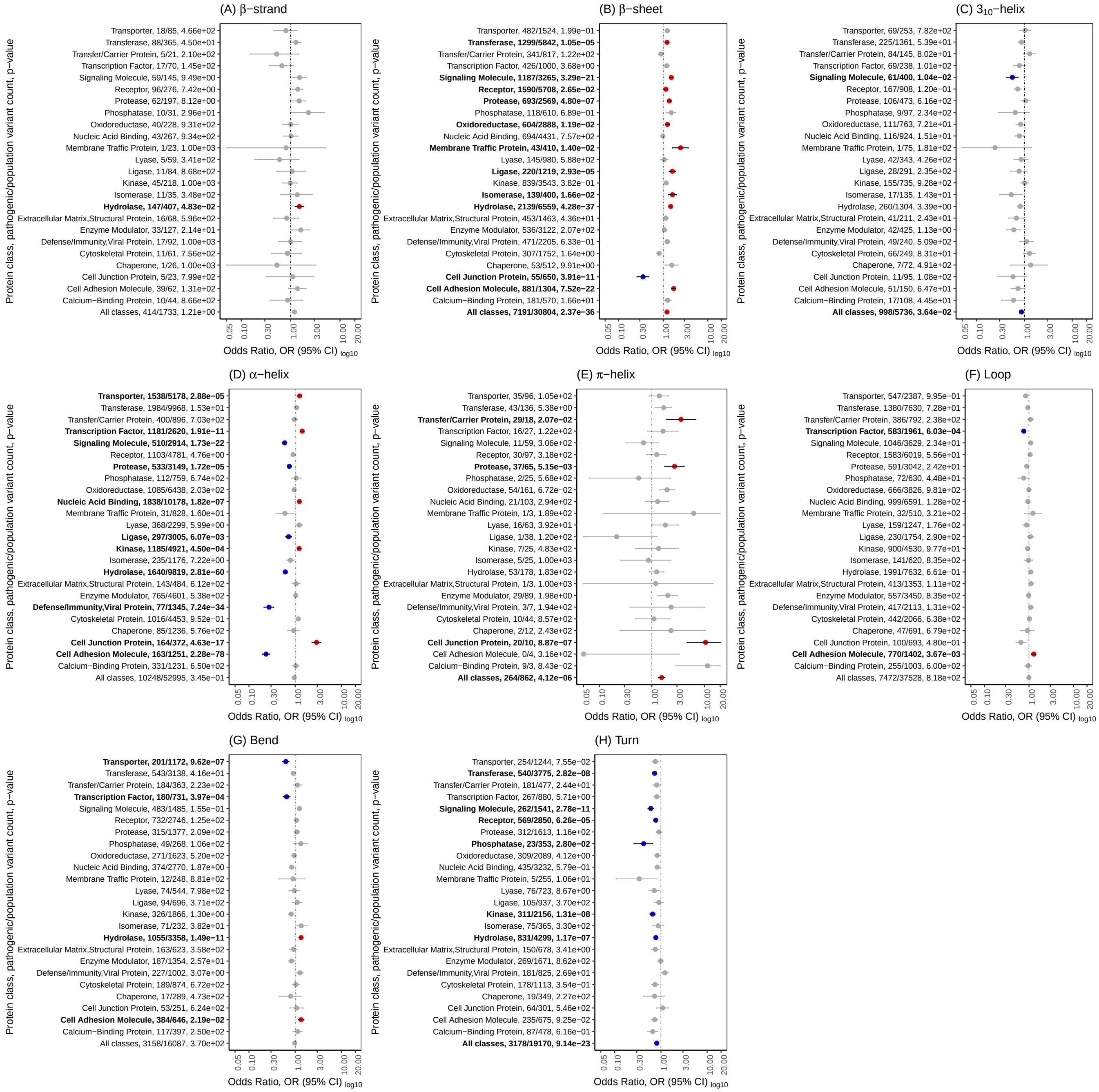

Fig. S3. Enrichment of pathogenic and population missense variants for "All classes" and twenty-four protein functional-classes in the eight-class secondary structure types. The plot shows the results of two-sided Fisher's exact tests of association. Circles show the odds ratio (OR) and the horizontal bars show the 95% confidence interval (CI). The y-axis is labelled with the protein class name, counts of pathogenic and population variants with the corresponding feature, and corrected p or "q" values, showing the significance of the association (a value of 1.0e-297 is should be read as <1.0e-297, indicating maximum significance). The OR $>$ 1 and OR $<$ 1, along with $q <$ 0.05, indicate that the corresponding feature (plot label) has an enrichment of pathogenic (red circle) and population (blue circle) variants, for the respective protein class (y-axis). The OR = 1 (dashed vertical line) indicates that there is no association between a variant type (pathogenic or population) and a feature. To facilitate the visualization, minimum and maximum values of OR along x-axis are set to 0.05 and 20.0, respectively. For non-significant association ($q \geq$ 0.05), the circle and CI bar are grey.

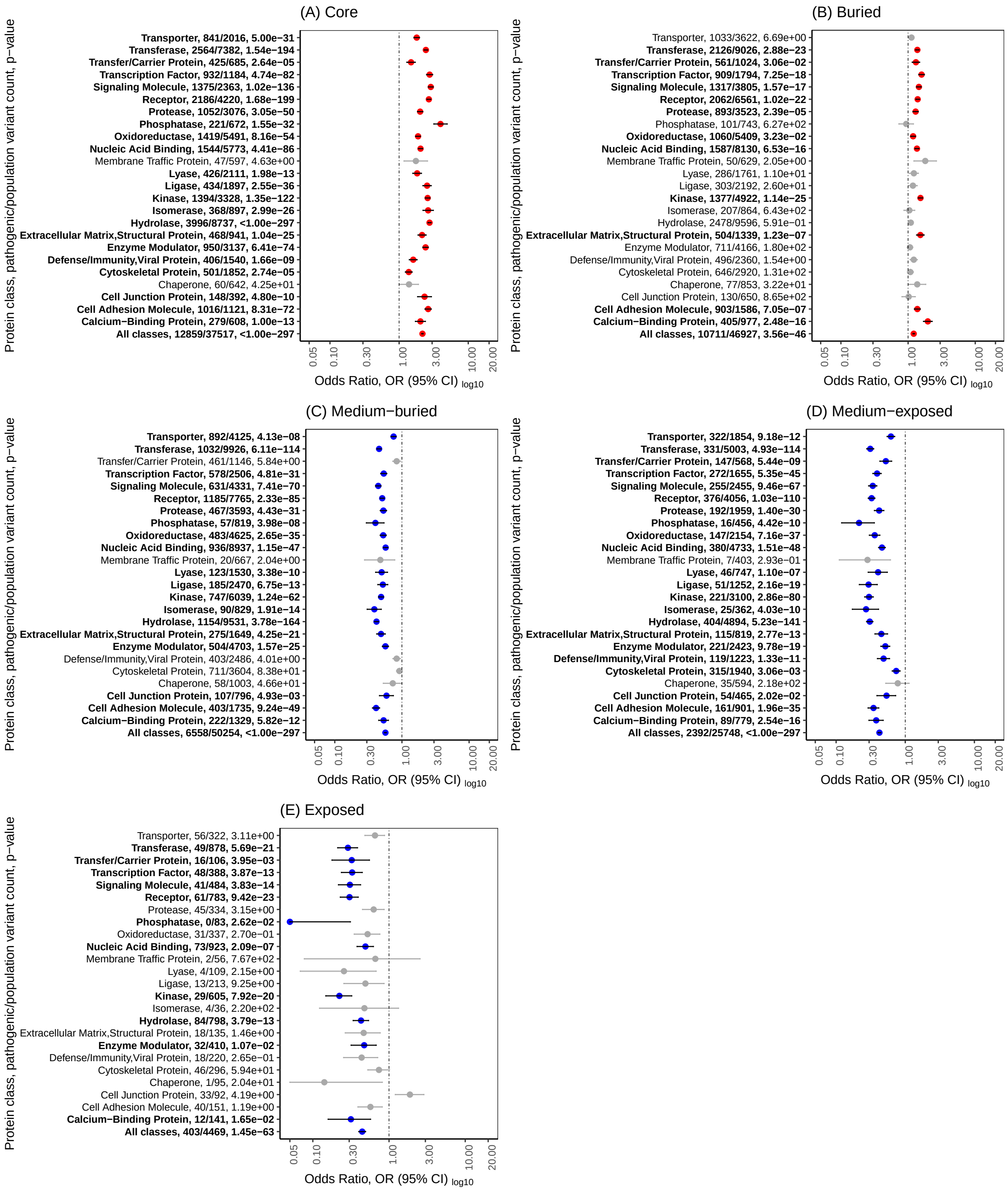

Fig. S4. Enrichment of pathogenic and population missense variants for "All classes" and twenty-four protein functional-classes in amino acid residues with different exposure levels. The plot shows the results of two-sided Fisher's exact tests of association. Circles show the odds ratio (OR) and the horizontal bars show the 95% confidence interval (CI). The y-axis ticks are labelled with the protein class name, counts of pathogenic and population variants with the corresponding feature, and corrected p or "q" values, showing the significance of the association (a value of 1.0e-297 is should be read as <1.0e-297, indicating maximum significance). The OR $>$ 1 and OR $<$ 1, along with $q <$ 0.05, indicate that the corresponding feature (plot label) has an enrichment of pathogenic (red circle) and population (blue circle) variants, for the respective protein class (y-axis). The OR = 1 (dashed vertical line) indicates that there is no association between a variant type (pathogenic or population) and a feature. To facilitate the visualization, minimum and maximum values of OR along x-axis are set to 0.05 and 20.0, respectively. For non-significant association ($q \geq$ 0.05), the circle and CI bar are grey.

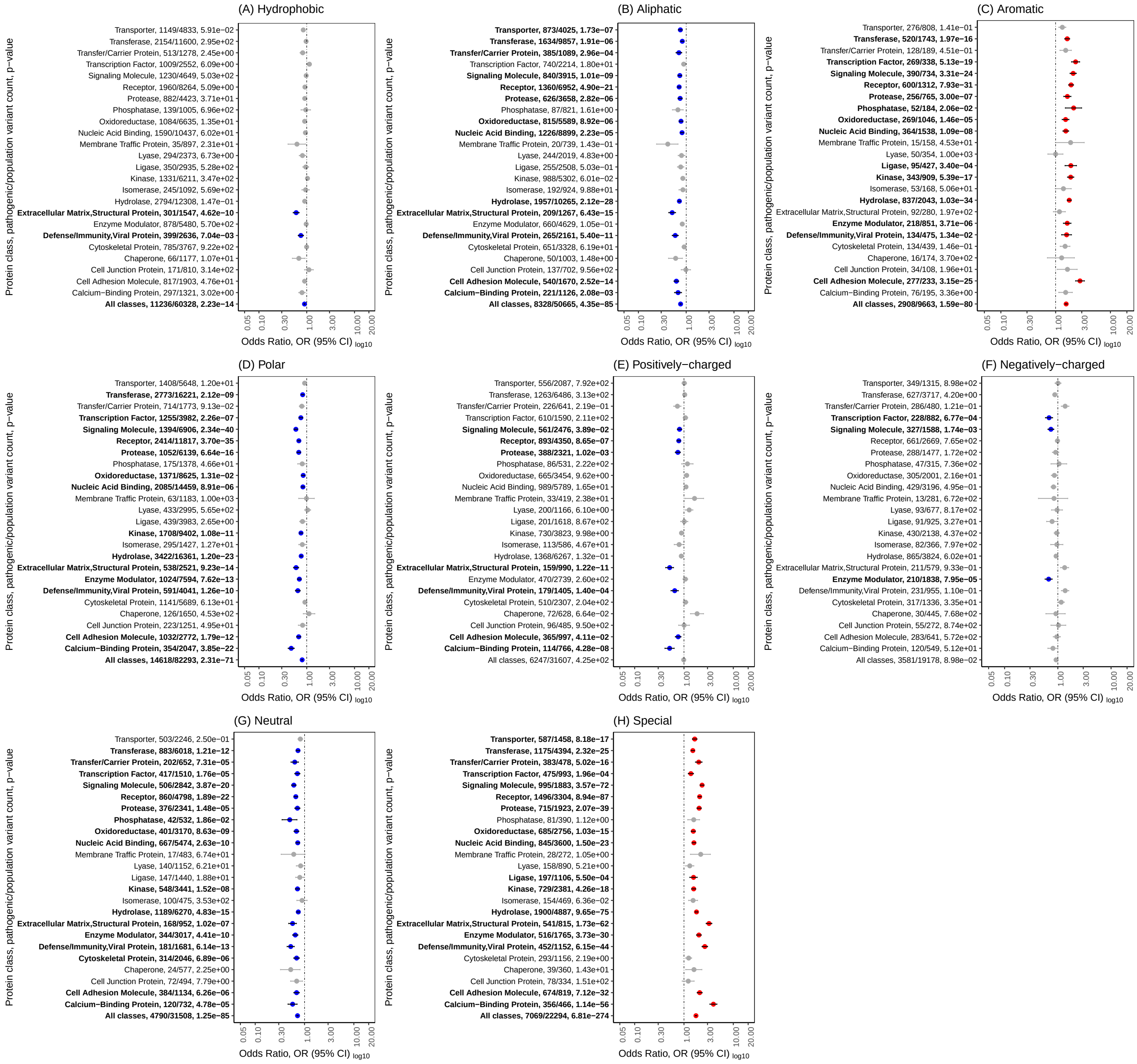

Fig. S5. Enrichment of pathogenic and population missense variants for "All classes" and twenty-four protein functional-classes in amino acids of eight different physicochemical properties. The plot shows the results of two-sided Fisher's exact tests of association. Circles show the odds ratio (OR) and the horizontal bars show the 95% confidence interval (CI). The y-axis ticks are labelled with the protein class name, counts of pathogenic and population variants with the corresponding feature, and corrected p or "q" values, showing the significance of the association (a value of 1.0e-297 is should be read as <1.0e-297, indicating maximum significance). The OR $>$ 1 and OR $<$ 1, along with $q <$ 0.05, indicate that the corresponding feature (plot label) has an enrichment of pathogenic (red circle) and population (blue circle) variants, for the respective protein class (y-axis). The OR = 1 (dashed vertical line) indicates that there is no association between a variant type (pathogenic or population) and a feature. To facilitate the visualization, minimum and maximum values of OR along x-axis are set to 0.05 and 20.0, respectively. For non-significant association ($q \geq$ 0.05), the circle and CI bar are grey.

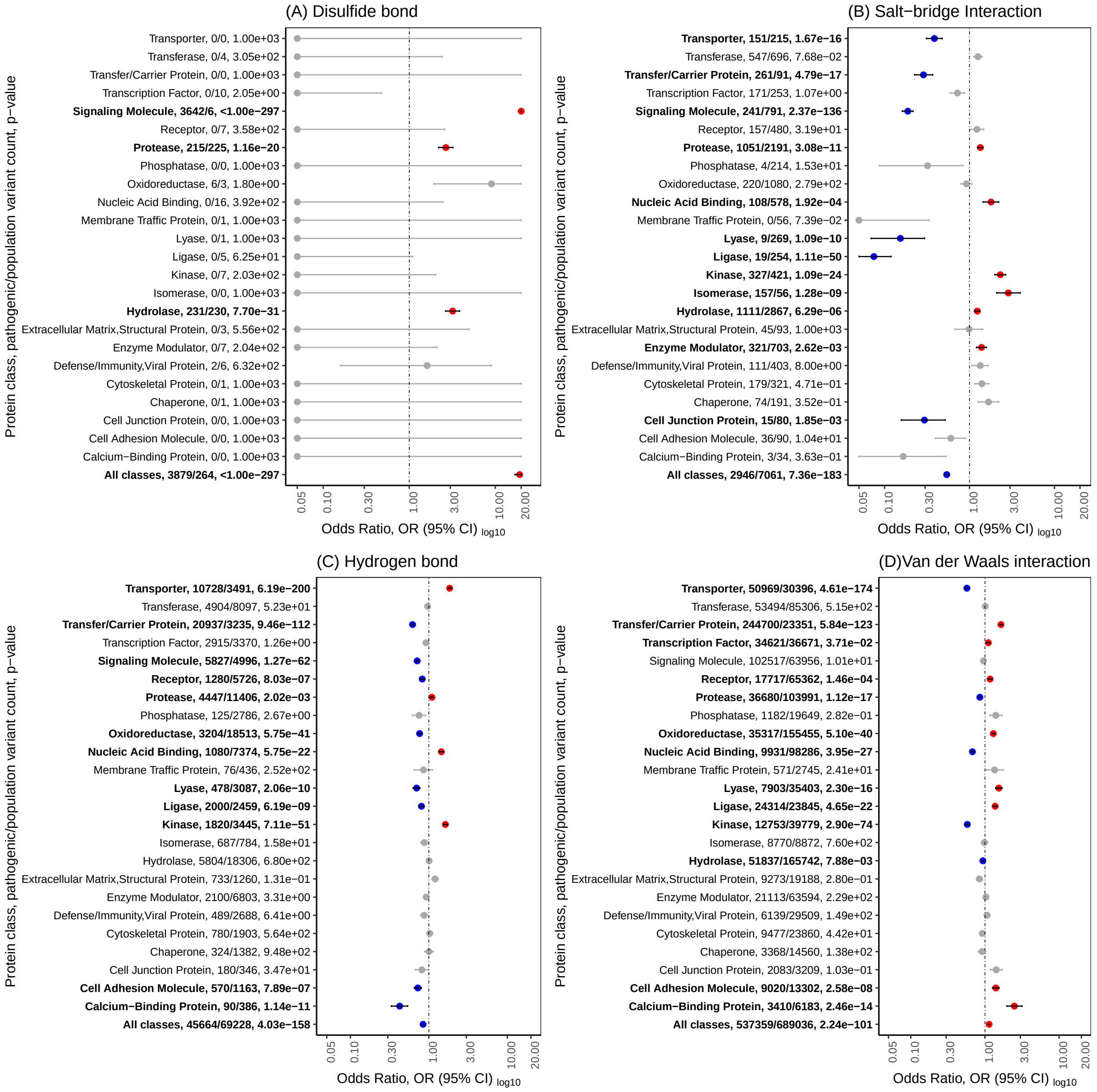

Fig. S6. Enrichment of pathogenic and population missense variants for “All classes” and twenty-four protein functional-classes in protein-protein interaction types. The plot shows the results of two-sided Fisher’s exact tests of association. Circles show the odds ratio (OR) and the horizontal bars show the 95% confidence interval (CI). The y-axis ticks are labelled with the protein class name, counts of pathogenic and population variants with the corresponding feature, and corrected p or “q” values, showing the significance of the association (a value of 1.0e-297 is should be read as <1.0e-297, indicating maximum significance). The OR $>$ 1 and OR $<$ 1, along with $q <$ 0.05, indicate that the corresponding feature (plot label) has an enrichment of pathogenic (red circle) and population (blue circle) variants, for the respective protein class (y-axis). The OR = 1 (dashed vertical line) indicates that there is no association between a variant type (pathogenic or population) and a feature. To facilitate the visualization, minimum and maximum values of OR along x-axis are set to 0.05 and 20.0, respectively. For non-significant association ($q \geq$ 0.05), the circle and CI bar are grey.

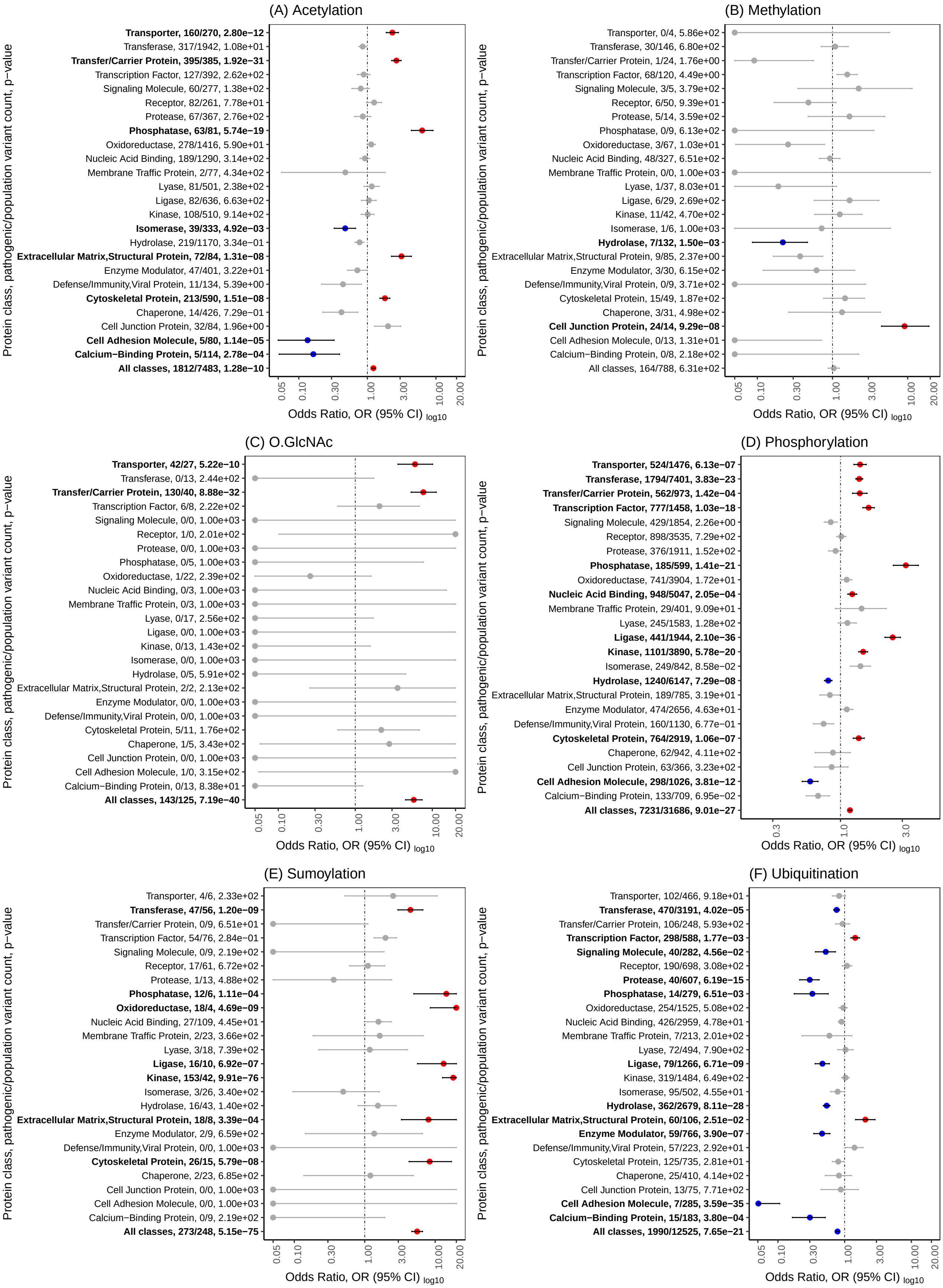
(A) Acetylation
Transporter, 160/270, 2.80e−12
Transferase, 317/1942, 1.08e+01
Transfer/Carrier Protein, 395/385, 1.92e−31
Transcription Factor, 127/392, 2.62e+02
Signaling Molecule, 60/277, 1.38e+02
Receptor, 82/261, 7.78e+01
Protease, 67/367, 2.76e+02
Phosphatase, 63/81, 5.74e−19
Oxidoreductase, 278/1416, 5.90e+01
Nucleic Acid Binding, 189/1290, 3.14e+02
Membrane Traffic Protein, 2/77, 4.34e+02
Lyase, 81/501, 2.38e+02
Ligase, 82/636, 6.63e+02
Kinase, 108/510, 9.14e+02
Isomerase, 39/333, 4.92e−03
Hydrolase, 219/1170, 3.34e−01
Extracellular Matrix,Structural Protein, 72/84, 1.31e−08
Enzyme Modulator, 47/401, 3.22e+01
Defense/Immunity,Viral Protein, 11/134, 5.39e+00
Cytoskeletal Protein, 213/590, 1.51e−08
Chaperone, 14/426, 7.29e−01
Cell Junction Protein, 32/84, 1.96e+00
Cell Adhesion Molecule, 5/80, 1.14e−05
Calcium−Binding Protein, 5/114, 2.78e−04
All classes, 1812/7483, 1.28e−10
Protein class, pathogenic/population variant count, p−value
0.05 0.10 0.30 1.00 3.00 10.00 20.00
Odds Ratio, OR (95% CI) log10
(B) Methylation
Transporter, 0/4, 5.86e+02
Transferase, 30/146, 6.80e+02
Transfer/Carrier Protein, 1/24, 1.76e+00
Transcription Factor, 68/120, 4.49e+00
Signaling Molecule, 3/5, 3.79e+02
Receptor, 6/50, 9.39e+01
Protease, 5/14, 3.59e+02
Phosphatase, 0/9, 6.13e+02
Oxidoreductase, 3/67, 1.03e+01
Nucleic Acid Binding, 48/327, 6.51e+02
Membrane Traffic Protein, 0/0, 1.00e+03
Lyase, 1/37, 8.03e+01
Ligase, 6/29, 2.69e+02
Kinase, 11/42, 4.70e+02
Isomerase, 1/6, 1.00e+03
Hydrolase, 7/132, 1.50e−03
Extracellular Matrix,Structural Protein, 9/85, 2.37e+00
Enzyme Modulator, 3/30, 6.15e+02
Defense/Immunity,Viral Protein, 0/9, 3.71e+02
Cytoskeletal Protein, 15/49, 1.87e+02
Chaperone, 3/31, 4.98e+02
Cell Junction Protein, 24/14, 9.29e−08
Cell Adhesion Molecule, 0/13, 1.31e+01
Calcium−Binding Protein, 0/8, 2.18e+02
All classes, 164/788, 6.31e+02
Protein class, pathogenic/population variant count, p−value
0.05 0.10 0.30 1.00 3.00 10.00 20.00
Odds Ratio, OR (95% CI) log10
(C) O.GlcNAc
Transporter, 42/27, 5.22e−10
Transferase, 0/13, 2.44e+02
Transfer/Carrier Protein, 130/40, 8.88e−32
Transcription Factor, 6/8, 2.22e+02
Signaling Molecule, 0/0, 1.00e+03
Receptor, 1/0, 2.01e+02
Protease, 0/0, 1.00e+03
Phosphatase, 0/5, 1.00e+03
Oxidoreductase, 1/22, 2.39e+02
Nucleic Acid Binding, 0/3, 1.00e+03
Membrane Traffic Protein, 0/3, 1.00e+03
Lyase, 0/17, 2.56e+02
Ligase, 0/0, 1.00e+03
Kinase, 0/13, 1.43e+02
Isomerase, 0/0, 1.00e+03
Hydrolase, 0/5, 5.91e+02
Extracellular Matrix,Structural Protein, 2/2, 2.13e+02
Enzyme Modulator, 0/0, 1.00e+03
Defense/Immunity,Viral Protein, 0/0, 1.00e+03
Cytoskeletal Protein, 5/11, 1.76e+02
Chaperone, 1/5, 3.43e+02
Cell Junction Protein, 0/0, 1.00e+03
Cell Adhesion Molecule, 1/0, 3.15e+02
Calcium−Binding Protein, 0/13, 8.38e+01
All classes, 143/125, 7.19e−40
Protein class, pathogenic/population variant count, p−value
0.05 0.10 0.30 1.00 3.00 10.00 20.00
Odds Ratio, OR (95% CI) log10
(D) Phosphorylation
Transporter, 524/1476, 6.13e−07
Transferase, 1794/7401, 3.83e−23
Transfer/Carrier Protein, 562/973, 1.42e−04
Transcription Factor, 777/1458, 1.03e−18
Signaling Molecule, 429/1854, 2.26e+00
Receptor, 898/3535, 7.29e+02
Protease, 376/1911, 1.52e+02
Phosphatase, 185/599, 1.41e−21
Oxidoreductase, 741/3904, 1.72e+01
Nucleic Acid Binding, 948/5047, 2.05e−04
Membrane Traffic Protein, 29/401, 9.09e+01
Lyase, 245/1583, 1.28e+02
Ligase, 441/1944, 2.10e−36
Kinase, 1101/3890, 5.78e−20
Isomerase, 249/842, 8.58e−02
Hydrolase, 1240/6147, 7.29e−08
Extracellular Matrix,Structural Protein, 189/785, 3.19e+01
Enzyme Modulator, 474/2656, 4.63e+01
Defense/Immunity,Viral Protein, 160/1130, 6.77e−01
Cytoskeletal Protein, 764/2919, 1.06e−07
Chaperone, 62/942, 4.11e+02
Cell Junction Protein, 63/366, 3.23e+02
Cell Adhesion Molecule, 298/1026, 3.81e−12
Calcium−Binding Protein, 133/709, 6.95e−02
All classes, 7231/31686, 9.01e−27
Protein class, pathogenic/population variant count, p−value
0.3 1.0 3.0
Odds Ratio, OR (95% CI) log10
(E) Sumoylation
Transporter, 4/6, 2.33e+02
Transferase, 47/56, 1.20e−09
Transfer/Carrier Protein, 0/9, 6.51e+01
Transcription Factor, 54/76, 2.84e−01
Signaling Molecule, 0/9, 2.19e+02
Receptor, 17/61, 6.72e+02
Protease, 1/13, 4.88e+02
Phosphatase, 12/6, 1.11e−04
Oxidoreductase, 18/4, 4.69e−09
Nucleic Acid Binding, 27/109, 4.45e+01
Membrane Traffic Protein, 2/23, 3.66e+02
Lyase, 3/18, 7.39e+02
Ligase, 16/10, 6.92e−07
Kinase, 153/42, 9.91e−76
Isomerase, 3/26, 3.40e+02
Hydrolase, 16/43, 1.40e+02
Extracellular Matrix,Structural Protein, 18/8, 3.39e−04
Enzyme Modulator, 2/9, 6.59e+02
Defense/Immunity,Viral Protein, 0/0, 1.00e+03
Cytoskeletal Protein, 26/15, 5.79e−08
Chaperone, 2/23, 6.85e+02
Cell Junction Protein, 0/0, 1.00e+03
Cell Adhesion Molecule, 0/0, 1.00e+03
Calcium−Binding Protein, 0/9, 2.19e+02
All classes, 273/248, 5.15e−75
Protein class, pathogenic/population variant count, p−value
0.05 0.10 0.30 1.00 3.00 10.00 20.00
Odds Ratio, OR (95% CI) log10
(F) Ubiquitination
Transporter, 102/466, 9.18e+01
Transferase, 470/3191, 4.02e−05
Transfer/Carrier Protein, 106/248, 5.93e+02
Transcription Factor, 298/588, 1.77e−03
Signaling Molecule, 40/282, 4.56e−02
Receptor, 190/698, 3.08e+02
Protease, 40/607, 6.19e−15
Phosphatase, 14/279, 6.51e−03
Oxidoreductase, 254/1525, 5.08e+02
Nucleic Acid Binding, 426/2959, 4.78e+01
Membrane Traffic Protein, 7/213, 2.01e+02
Lyase, 72/494, 7.90e+02
Ligase, 79/1266, 6.71e−09
Kinase, 319/1484, 6.49e+02
Isomerase, 95/502, 4.55e+01
Hydrolase, 362/2679, 8.11e−28
Extracellular Matrix,Structural Protein, 60/106, 2.51e−02
Enzyme Modulator, 59/766, 3.90e−07
Defense/Immunity,Viral Protein, 57/223, 2.92e+01
Cytoskeletal Protein, 125/735, 2.81e+01
Chaperone, 25/410, 4.14e+02
Cell Junction Protein, 13/75, 7.71e+02
Cell Adhesion Molecule, 7/285, 3.59e−35
Calcium−Binding Protein, 15/183, 3.80e−04
All classes, 1990/12525, 7.65e−21
Protein class, pathogenic/population variant count, p−value
0.05 0.10 0.30 1.00 3.00 10.00 20.00
Odds Ratio, OR (95% CI) log10

Fig. S7

Fig. S7. (Previous page.) Enrichment of pathogenic and population missense variants for “All classes” and twenty-four protein functional-classes in post-translational modification types. The plot shows the results of two-sided Fisher’s exact tests of association. Circles show the odds ratio (OR) and the horizontal bars show the 95% confidence interval (CI). The y-axis ticks are labelled with the protein class name, counts of pathogenic and population variants with the corresponding feature, and corrected p or “q” values, showing the significance of the association (a value of 1.0e-297 is should be read as <1.0e-297, indicating maximum significance). The OR $>$ 1 and OR $<$ 1, along with $q <$ 0.05, indicate that the corresponding feature (plot label) has an enrichment of pathogenic (red circle) and population (blue circle) variants, for the respective protein class (y-axis). The OR = 1 (dashed vertical line) indicates that there is no association between a variant type (pathogenic or population) and a feature. To facilitate the visualization, minimum and maximum values of OR along x-axis are set to 0.05 and 20.0, respectively. For non-significant association ($q \geq$ 0.05), the circle and CI bar are grey.

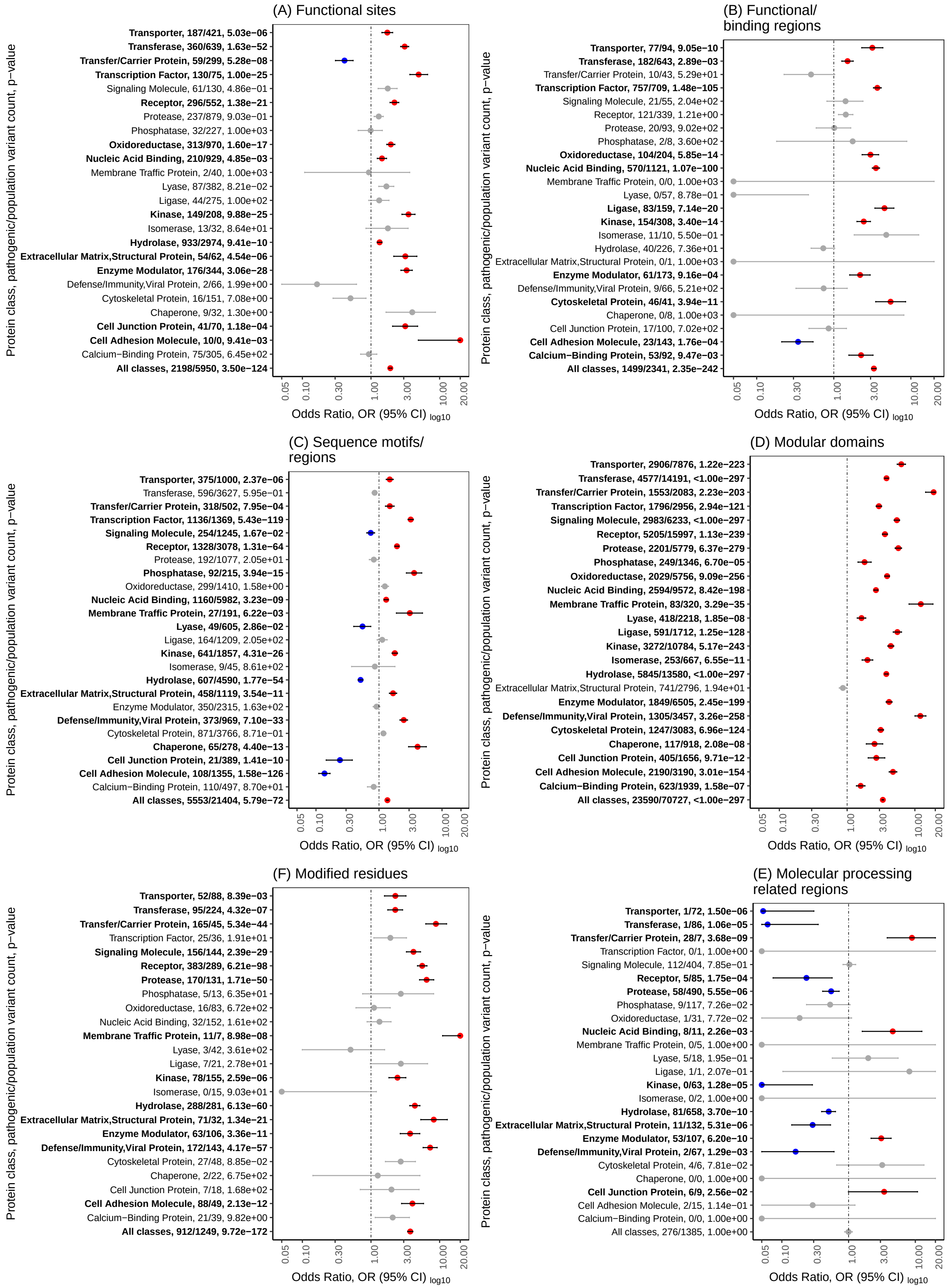
(A) Functional sites
Transporter, 187/421, 5.03e−06
Transferase, 360/639, 1.63e−52
Transfer/Carrier Protein, 59/299, 5.28e−08
Transcription Factor, 130/75, 1.00e−25
Signaling Molecule, 61/130, 4.86e−01
Receptor, 296/552, 1.38e−21
Protease, 237/879, 9.03e−01
Phosphatase, 32/227, 1.00e+03
Oxidoreductase, 313/970, 1.60e−17
Nucleic Acid Binding, 210/929, 4.85e−03
Membrane Traffic Protein, 2/40, 1.00e+03
Lyase, 87/382, 8.21e−02
Ligase, 44/275, 1.00e+02
Kinase, 149/208, 9.88e−25
Isomerase, 13/32, 8.64e+01
Hydrolase, 933/2974, 9.41e−10
Extracellular Matrix,Structural Protein, 54/62, 4.54e−06
Enzyme Modulator, 176/344, 3.06e−28
Defense/Immunity,Viral Protein, 2/66, 1.99e+00
Cytoskeletal Protein, 16/151, 7.08e+00
Chaperone, 9/32, 1.30e+00
Cell Junction Protein, 41/70, 1.18e−04
Cell Adhesion Molecule, 10/0, 9.41e−03
Calcium−Binding Protein, 75/305, 6.45e+02
All classes, 2198/5950, 3.50e−124
(B) Functional/ binding regions
Transporter, 77/94, 9.05e−10
Transferase, 182/643, 2.89e−03
Transfer/Carrier Protein, 10/43, 5.29e+01
Transcription Factor, 757/709, 1.48e−105
Signaling Molecule, 21/55, 2.04e+02
Receptor, 121/339, 1.21e+00
Protease, 20/93, 9.02e+02
Phosphatase, 2/8, 3.60e+02
Oxidoreductase, 104/204, 5.85e−14
Nucleic Acid Binding, 570/1121, 1.07e−100
Membrane Traffic Protein, 0/0, 1.00e+03
Lyase, 0/57, 8.78e−01
Ligase, 83/159, 7.14e−20
Kinase, 154/308, 3.40e−14
Isomerase, 11/10, 5.50e−01
Hydrolase, 40/226, 7.36e+01
Extracellular Matrix,Structural Protein, 0/1, 1.00e+03
Enzyme Modulator, 61/173, 9.16e−04
Defense/Immunity,Viral Protein, 9/66, 5.21e+02
Cytoskeletal Protein, 46/41, 3.94e−11
Chaperone, 0/8, 1.00e+03
Cell Junction Protein, 17/100, 7.02e+02
Cell Adhesion Molecule, 23/143, 1.76e−04
Calcium−Binding Protein, 53/92, 9.47e−03
All classes, 1499/2341, 2.35e−242
(C) Sequence motifs/ regions
Transporter, 375/1000, 2.37e−06
Transferase, 596/3627, 5.95e−01
Transfer/Carrier Protein, 318/502, 7.95e−04
Transcription Factor, 1136/1369, 5.43e−119
Signaling Molecule, 254/1245, 1.67e−02
Receptor, 1328/3078, 1.31e−64
Protease, 192/1077, 2.05e+01
Phosphatase, 92/215, 3.94e−15
Oxidoreductase, 299/1410, 1.58e+00
Nucleic Acid Binding, 1160/5982, 3.23e−09
Membrane Traffic Protein, 27/191, 6.22e−03
Lyase, 49/605, 2.86e−02
Ligase, 164/1209, 2.05e+02
Kinase, 641/1857, 4.31e−26
Isomerase, 9/45, 8.61e+02
Hydrolase, 607/4590, 1.77e−54
Extracellular Matrix,Structural Protein, 458/1119, 3.54e−11
Enzyme Modulator, 350/2315, 1.63e+02
Defense/Immunity,Viral Protein, 373/969, 7.10e−33
Cytoskeletal Protein, 871/3766, 8.71e−01
Chaperone, 65/278, 4.40e−13
Cell Junction Protein, 21/389, 1.41e−10
Cell Adhesion Molecule, 108/1355, 1.58e−126
Calcium−Binding Protein, 110/497, 8.70e+01
All classes, 5553/21404, 5.79e−72
(D) Modular domains
Transporter, 2906/7876, 1.22e−223
Transferase, 4577/14191, <1.00e−297
Transfer/Carrier Protein, 1553/2083, 2.23e−203
Transcription Factor, 1796/2956, 2.94e−121
Signaling Molecule, 2983/6233, <1.00e−297
Receptor, 5205/15997, 1.13e−239
Protease, 2201/5779, 6.37e−279
Phosphatase, 249/1346, 6.70e−05
Oxidoreductase, 2029/5756, 9.09e−256
Nucleic Acid Binding, 2594/9572, 8.42e−198
Membrane Traffic Protein, 83/320, 3.29e−35
Lyase, 418/2218, 1.85e−08
Ligase, 591/1712, 1.25e−128
Kinase, 3272/10784, 5.17e−243
Isomerase, 253/667, 6.55e−11
Hydrolase, 5845/13580, <1.00e−297
Extracellular Matrix,Structural Protein, 741/2796, 1.94e+01
Enzyme Modulator, 1849/6505, 2.45e−199
Defense/Immunity,Viral Protein, 1305/3457, 3.26e−258
Cytoskeletal Protein, 1247/3083, 6.96e−124
Chaperone, 117/918, 2.08e−08
Cell Junction Protein, 405/1656, 9.71e−12
Cell Adhesion Molecule, 2190/3190, 3.01e−154
Calcium−Binding Protein, 623/1939, 1.58e−07
All classes, 23590/70727, <1.00e−297
(F) Modified residues
Transporter, 52/88, 8.39e−03
Transferase, 95/224, 4.32e−07
Transfer/Carrier Protein, 165/45, 5.34e−44
Transcription Factor, 25/36, 1.91e+01
Signaling Molecule, 156/144, 2.39e−29
Receptor, 383/289, 6.21e−98
Protease, 170/131, 1.71e−50
Phosphatase, 5/13, 6.35e+01
Oxidoreductase, 16/83, 6.72e+02
Nucleic Acid Binding, 32/152, 1.61e+02
Membrane Traffic Protein, 11/7, 8.98e−08
Lyase, 3/42, 3.61e+02
Ligase, 7/21, 2.78e+01
Kinase, 78/155, 2.59e−06
Isomerase, 0/15, 9.03e+01
Hydrolase, 288/281, 6.13e−60
Extracellular Matrix,Structural Protein, 71/32, 1.34e−21
Enzyme Modulator, 63/106, 3.36e−11
Defense/Immunity,Viral Protein, 172/143, 4.17e−57
Cytoskeletal Protein, 27/48, 8.85e−02
Chaperone, 2/22, 6.75e+02
Cell Junction Protein, 7/18, 1.68e+02
Cell Adhesion Molecule, 88/49, 2.13e−12
Calcium−Binding Protein, 21/39, 9.82e+00
All classes, 912/1249, 9.72e−172
(E) Molecular processing related regions
Transporter, 1/72, 1.50e−06
Transferase, 1/86, 1.06e−05
Transfer/Carrier Protein, 28/7, 3.68e−09
Transcription Factor, 0/1, 1.00e+00
Signaling Molecule, 112/404, 7.85e−01
Receptor, 5/85, 1.75e−04
Protease, 58/490, 5.55e−06
Phosphatase, 9/117, 7.26e−02
Oxidoreductase, 1/31, 7.72e−02
Nucleic Acid Binding, 8/11, 2.26e−03
Membrane Traffic Protein, 0/5, 1.00e+00
Lyase, 5/18, 1.95e−01
Ligase, 1/1, 2.07e−01
Kinase, 0/63, 1.28e−05
Isomerase, 0/2, 1.00e+00
Hydrolase, 81/658, 3.70e−10
Extracellular Matrix,Structural Protein, 11/132, 5.31e−06
Enzyme Modulator, 53/107, 6.20e−10
Defense/Immunity,Viral Protein, 2/67, 1.29e−03
Cytoskeletal Protein, 4/6, 7.81e−02
Chaperone, 0/0, 1.00e+00
Cell Junction Protein, 6/9, 2.56e−02
Cell Adhesion Molecule, 2/15, 1.14e−01
Calcium−Binding Protein, 0/0, 1.00e+00
All classes, 276/1385, 1.00e+00
Protein class, pathogenic/population variant count, p−value
Odds Ratio, OR (95% CI) log10
0.05
0.10
0.30
1.00
3.00
10.00
20.00

Fig. S8

Fig. S8. (Previous page.) Enrichment of pathogenic and population missense variants for “All classes” and twenty-four protein functional-classes in six UniProt-based functional features. The plot shows the results of two-sided Fisher’s exact tests of association. Circles show the odds ratio (OR) and the horizontal bars show the 95% confidence interval (CI). The y-axis ticks are labelled with the protein class name, counts of pathogenic and population variants with the corresponding feature, and corrected p or “q” values, showing the significance of the association (a value of 1.0e-297 is should be read as <1.0e-297, indicating maximum significance). The OR $>$ 1 and OR $<$ 1, along with $q <$ 0.05, indicate that the corresponding feature (plot label) has an enrichment of pathogenic (red circle) and population (blue circle) variants, for the respective protein class (y-axis). The OR = 1 (dashed vertical line) indicates that there is no association between a variant type (pathogenic or population) and a feature. To facilitate the visualization, minimum and maximum values of OR along x-axis are set to 0.05 and 20.0, respectively. For non-significant association ($q \geq$ 0.05), the circle and CI bar are grey.

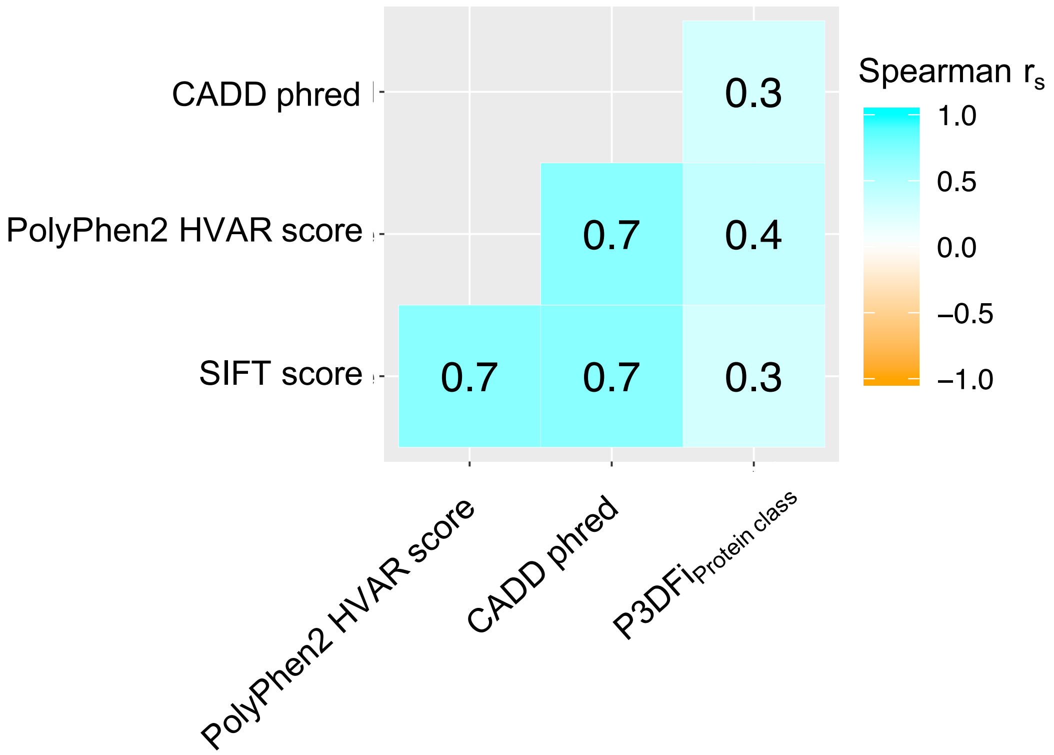

Fig. S9. Pairwise correlation (Spearman's non-parametric test) between the protein class specific pathogenic 3D Feature index ($P3DFi_{Protein\ class}$) derived in this study and the three existing variant pathogenicity prediction scores: SIFT (22), PolyPhen2 (23) and CADD (24). Note that, unlike the other scores P3DFi was not generated by a learning model to predict pathogenicity, instead it quantifies the difference between the pathogenic and population variant associated 3D features for each amino acid. P3DFi > 0 thus indicates the 3D mutational hotspots. The correlation plot shows that P3DFi has the maximum correlation of only 40% with the PolyPhen2 score. We thus speculate that P3DFi, derived using the 3D structure related information only, can serve as an orthogonal determinant of pathogenicity when combined with these existing methods (see **Table 1** and **Fig. 5** in the main text for relevant results).

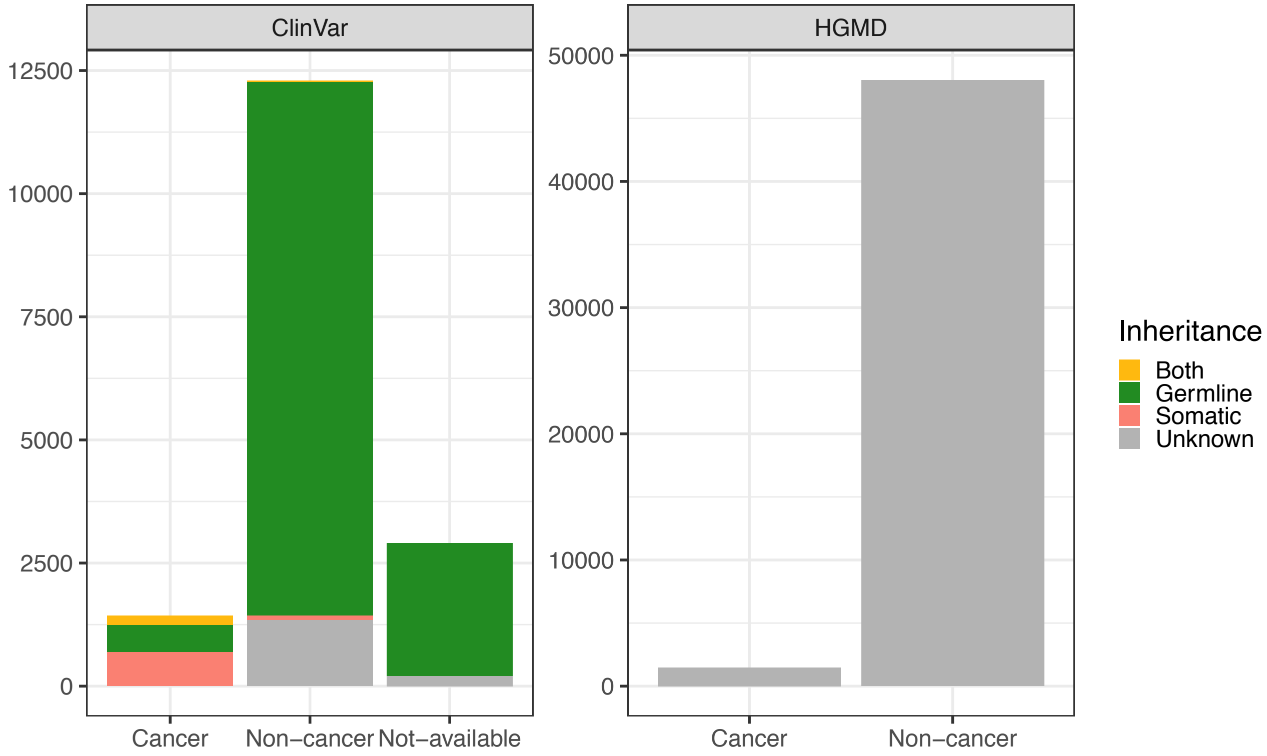

Fig. S10. Distribution of pathogenic variations in 1,330 genes (DAGS1330 set, analyzed in this work) associated with their respective phenotype: cancer, non-cancer and not-provided, and according to the variants' allele origin or inheritance, that is: somatic, germline, both and unknown (as available in the ClinVar database (7)). Variants were considered to be related to cancer if the phenotype description included at least one of the following keywords: "cancer", "carcinoma", "leukemia", "tumor", "neoplasm", "sarcoma", "melanoma", "lynphoma" or "glioma". "Not provided" and/or "Unknown" phenotypes were kept separate as "Not-provided". Out of the 16,638 pathogenic and likely pathogenic variations from ClinVar database (7), 74% (n = 12,301) are associated with non-cancer phenotype, 9% (n = 1,431) are associated with cancer, and for 17% (n = 2,906) the phenotype data are not available. At the same time, out of the 49,501 disease mutations from HGMD database (8), 97% (n = 48,013) are associated with non-cancer phenotype. In total, out of all pathogenic variants, 91% have non-cancer mutations and only about 4% have cancer mutations. Importantly, only about 1% of all variations in these 1,330 genes obtained from ClinVar and HGMD databases are somatic (assuming all HGMD variants are germline according to the literature (8)).

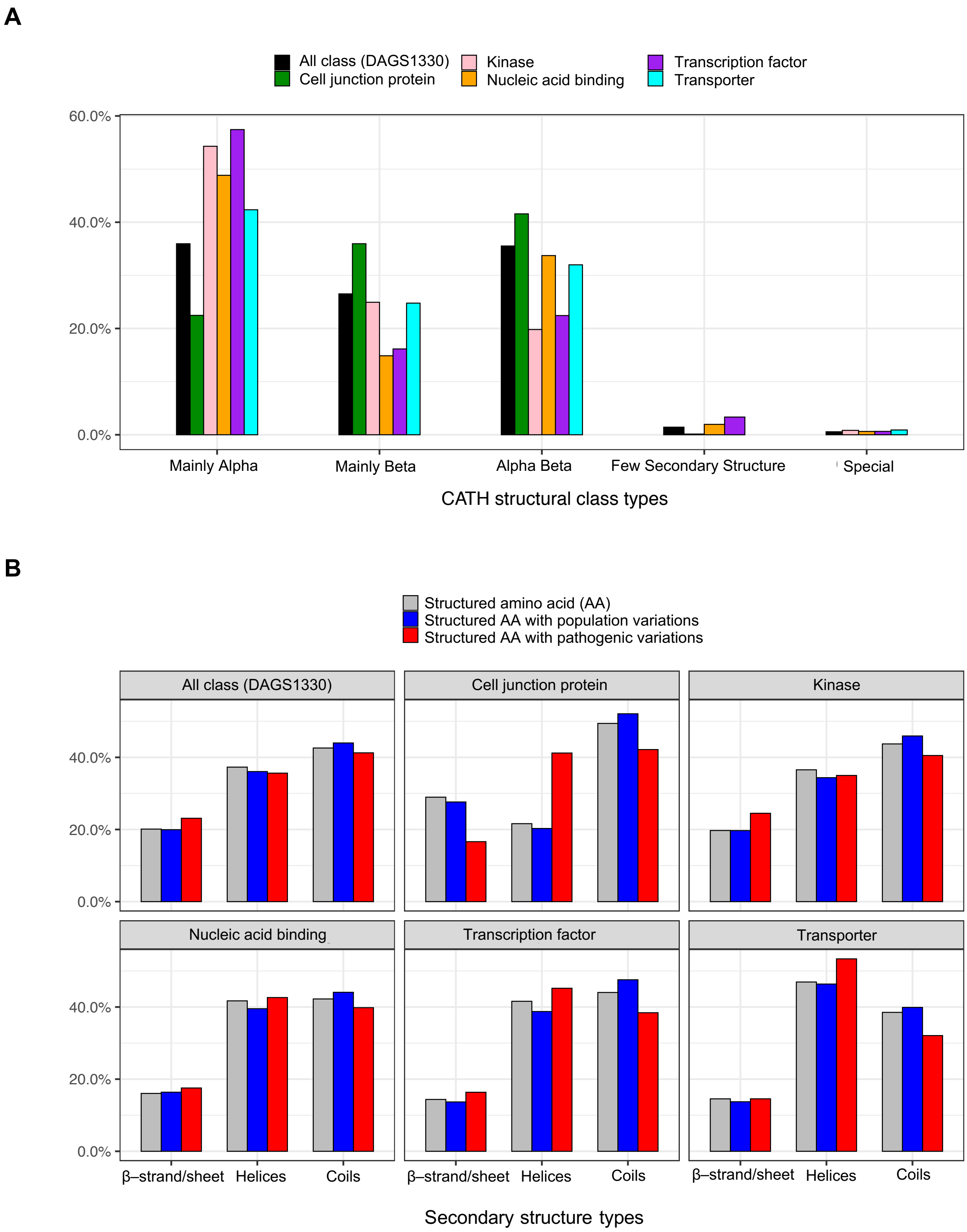

A
All class (DAGS1330)
Cell junction protein
Kinase
Nucleic acid binding
Transcription factor
Transporter
60.0%
40.0%
20.0%
0.0%
Mainly Alpha
Mainly Beta
Alpha Beta
Few Secondary Structure
Special
CATH structural class types
B
Structured amino acid (AA)
Structured AA with population variations
Structured AA with pathogenic variations
All class (DAGS1330)
Cell junction protein
Kinase
Nucleic acid binding
Transcription factor
Transporter
40.0%
20.0%
0.0%
β–strand/sheet
Helices
Coils
Secondary structure types

Fig. S11

Fig. S11. (Previous page.) Pathogenic variations were found enriched in α-helix for five protein classes: cell junction protein, kinase, nucleic acid binding, transcription factor, and transporter, in contrast to the "All class (DAGS1330)" set, because of a higher observed rate of pathogenic variations on α-helix than the population variations. (*A*) Distribution of different folds adopted by proteins from all classes and from the five aforementioned classes (collected from the CATH Protein Structural Classification database (27)). For 1,170 out of 1,330 proteins, CATH annotations were available (version: April 2020) for 5,649 unique chains in a structure from the PDB (1). (*B*) Proportion of all residues, residues mutated in pathogenic variants and population variants of different secondary structure types (β-strand/sheet, helices, and coils), for all classes and for the five above mentioned protein classes. We observed that a higher proportion of protein chains from all classes adopt a "mainly alpha" fold (~36%) than "mainly beta" fold (~26%) (in *A*). This is also the case for four out of these five protein classes (kinase, nucleic acid binding, transcription factor, and transporter) where we find an enrichment of pathogenic mutations in helices, meaning that the percentage of pathogenic mutations is higher in this structural motif than that of population mutations. Notably, though, even in cell junction proteins, which adopt a mainly beta fold (~37% beta vs. ~22% alpha), we find an enrichment of pathogenic (42%) over population (20%) mutations in helices (OR = 2.8, SI Appendix, Fig. S2B), especially α-helix (OR = 2.9, *SI Appendix*, Fig. S3D) and α-helix (OR = 10.5, *SI Appendix*, Fig. S3E). This exemplifies that our results are robust with respect to the structural composition of proteins and, more in general, to the abundance of a given feature (see *Materials and Methods*).

Table S1. Number of 3D features associated to pathogenic and population missense variants (referred to as the characteristic features identified in this study) for "All classes" by joint analysis of 1,330 genes in the DAGS1330 dataset and individually for twenty-four protein functional classes (class-specific features).

Protein class	Characteristic 3D features of **pathogenic** variants	Class-specific characteristic features of **pathogenic** variants outside the "All class" based features (1st row, 2nd column)*	Characteristic 3D features of **population** variants	Class-specific characteristic features of **population** variants outside the "All class" based features (1st row, 4th column)*	Fraction of unique 3D features specific for protein classes outside the "All class" based features (%) (1st row, 2nd and 4th columns)$^{+}$
All classes (DAGS1330)	**18**	n/a	**14**	n/a	n/a
Calcium-Binding Protein	6	0	10	2	12.5
Cell Adhesion Molecule	13	3	14	6	33.3
Cell Junction Protein	7	3	6	3	46.2
Chaperone	2	0	0	0	0
Cytoskeletal Protein	6	0	2	0	0
Defense/Immunity protein	7	1	8	2	20.0
Enzyme Modulator	9	2	7	1	18.8
Extracellular Matrix Protein	9	1	8	2	17.6
Hydrolase	12	3	15	6	33.3
Isomerase	5	1	3	1	25.0
Kinase	15	4	9	2	25.0
Ligase	10	0	7	0	0
Lyase	3	0	5	1	12.5
Membrane Traffic Protein	5	0	0	0	0
Nucleic Acid Binding	13	4	8	1	23.8
Oxidoreductase	11	0	6	0	0
Phosphatase	7	0	6	0	0
Protease	12	2	11	4	26.1
Receptor	11	0	10	1	4.8
Signaling Molecule	9	0	16	3	12.0
Transcription Factor	13	3	9	3	27.3
Transfer/Carrier Protein	12	1	7	1	10.5
Transferase	12	0	10	1	4.5
Transporter	13	3	7	3	30.0

* Number of unique class-specific features of pathogenic and population variants outside the **18** and **14** features found by analyzing all genes, respectively.
+ Fraction of unique (out of the total) protein class-specific pathogenic and population variant associated features.

Table S2. Number of genes, missense variants/amino acid substitutions, protein structure in different steps of Disease-Associated Genes with Structure, namely DAGS1330, dataset preparation

	Population variants	Pathogenic variants	
	gnomAD (missense)	ClinVar (likely/-pathogenic missense)	HGMD (disease mutation)
Genes with experimentally solved structure available for the encoded proteins	$5,724$	$1,466$	$1,673$
Missense variants of the genes with structure available for the encoded proteins	$1,485,579$	$16,570$	$47,036$
Amino acid substitutions mapped on the structure	$496,869$	$8,137$	$30,730$
Genes with missense variants mappable on the structure	$4,897$	**1,330***	
Protein structures onto which the variants were mapped	$29,870$	**14,270***	
Missense variants of DAGS1330 genes that were mapped on the structure	**164,915**	**32,923***	

* combined (unique) counts from HGMD and ClinVar databases.

Number of genes, variants, and protein structures included in the **DAGS1330** dataset to perform the statistical analysis is bold faced.

Table S3. Comparison of the most predictive P3DFi (P3DFi > 2 and P3DFi < -2) to three state-of-the-art predictors (SIFT (22), PolyPhen2 (23) and CADD (24)) in stratifying pathogenic variants from benign variants.

Method	Recall/ Sensitivity/ True Positive Rate	Selectivity/ Specificity/ True Negative Rate	Balanced Accuracy	Matthews Correlation Coefficient (MCC)	F1 Score	Precision	Fall-out/ False Positive Rate	Miss Rate/ False Negative Rate
$\text{P3DFi}_{\text{Protein class}}$	83.68%	**85.65%**	**84.66%**	**67.18%**	87.53%	**91.74%**	**14.35%**	16.32%
$\text{P3DFi}^{*}_{\text{DAGS1330}}$	79.58%	83.73%	81.66%	60.90%	84.61%	90.31%	16.27%	20.42%
SIFT (22)	90.54%	73.84%	82.19%	65.79%	**88.65%**	86.84%	26.16%	9.46%
PolyPhen2 (23)	88.28%	78.95%	83.62%	67.03%	88.58%	88.88%	21.05%	11.72%
CADD (24)	**95.56%**	61.08%	78.32%	63.08%	88.49%	82.40%	38.92%	**4.44%**

The best and second best score values in each metric are boldfaced and underlined, respectively.
The performances are evaluated on the variants (total count = $1,824$) with the highest and lowest P3DFi values (see **Fig. 4**, P3DFi > 2 and P3DFi < -2).
* See computation of P3DFi in *Materials and Methods*.

SI Dataset S1 (Dataset_S1.xlsx)

Information of the genes in DAGS1330 (disease-associated genes with structure) dataset. Gene name (count = 1,330), UniProt protein accession number, protein length, CCDS identifier, Ensemble gene identifier, and HGNC identifier.

SI Dataset S2 (Dataset_S2.xlsx)

Protein functional class information. Protein class name, number of genes and protein structures in each class, number of pathogenic and population variations mapped on structures for each class, and the list of genes annotated in each protein class.

SI Dataset S3 (Dataset_S3.xlsx)

Protein 3D features associated to pathogenic and population missense variants (identified in this study) for "All classes" (joint analysis of $1,330$ genes in the DAGS1330 dataset), and individually for twenty-four protein functional classes. The columns represent seven main feature categories and each cell outlines the 3D feature that has a significant burden of pathogenic and/or population variations (two-sided Fisher's exact test, corrected p-value or "q" < 0.05). The 'x' indicates that no feature of the corresponding category was significantly associated with pathogenic or population variants.

SI Dataset S4 (Dataset_S4.xlsx)

Training dataset to develop the "Random forest" ensemble models. The first column reports the true class labels (1: deleterious or pathogenic, -1: neutral or benign). The next three columns show the scores generated by the variant pathogenicity predictors: SIFT, PolyPhen2, and CADD. The all protein (DAGS1330 dataset, **Dataset S1**) based and protein functional class specific pathogenic 3D feature index ($\text{P3DFi}_{\text{DAGS1330}}$ and $\text{P3DFi}_{\text{Protein class}}$) derived in this study are reported in the last two columns. For $209,110$ variants ($31,913$ pathogenic and $177,197$ benign) in the DAGS1330 dataset, all the existing predictors' scores were available, and therefore, were used for training. Three Random forest classifiers (number of decision trees = 2000, evaluation function = "gini", depth of the trees = 10) were developed: two models were trained separately with the $\text{P3DFi}_{\text{DAGS1330}}$ and $\text{P3DFi}_{\text{Protein class}}$ values in addition to the scores from SIFT, PolyPhen2 and CADD, and the third model was trained without any P3DFi values (see **Table 1** and **Fig. 5** in the main text for relevant results).

SI Dataset S5 (Dataset_S5.xlsx)

Test dataset to evaluate the "Random forest" ensemble models and compare their outputs to SIFT, PolyPhen2, and CADD scores. The first column reports the true class labels (1: deleterious or pathogenic, -1: neutral or benign). The next three columns show the scores generated by the variant pathogenicity predictors: SIFT, PolyPhen2, and CADD. Pathogenic 3D feature indices ($\text{P3DFi}_{\text{DAGS1330}}$ and $\text{P3DFi}_{\text{Protein class}}$) derived in this study are reported in the fifth and sixth columns, respectively. For $22,362$ ($17,707$ pathogenic and $4,655$ benign) out of $22,695$ variants in the validation set, all the existing predictors' scores were available, and therefore, were used for evaluation (see **Table 1** and **Fig. 5** in the main text for relevant results). Predicted probability outputs (>0.5: deleterious or pathogenic, ≤ 0.5: neutral or benign) from three ensemble models developed in this study are given in the last three columns.

References

1. HM Berman, PE Bourne, J Westbrook, C Zardecki, The protein data bank in *Protein Structure.* (CRC Press), pp. 394–410 (2003).
2. R Apweiler, et al., Uniprot: the universal protein knowledgebase. *Nucleic acids research* **32**, D115–D119 (2004).
3. S Velankar, et al., Sifts: structure integration with function, taxonomy and sequences resource. *Nucleic acids research* **41**, D483–D489 (2012).
4. KJ Karczewski, et al., The mutational constraint spectrum quantified from variation in 141,456 humans. *Nature* **581**, 434–443 (2020).
5. P Danecek, et al., The variant call format and vcftools. *Bioinformatics* **27**, 2156–2158 (2011).
6. DR Zerbino, et al., Ensembl 2018. *Nucleic acids research* **46**, D754–D761 (2018).
7. MJ Landrum, et al., Clinvar: improving access to variant interpretations and supporting evidence. *Nucleic acids research* **46**, D1062–D1067 (2018).
8. PD Stenson, et al., The human gene mutation database: building a comprehensive mutation repository for clinical and molecular genetics, diagnostic testing and personalized genomic medicine. *Hum. genetics* **133**, 1–9 (2014).
9. TL Mighell, S Evans-Dutson, BJ O'Roak, A saturation mutagenesis approach to understanding pten lipid phosphatase activity and genotype-phenotype relationships. *The Am. J. Hum. Genet.* **102**, 943–955 (2018).
10. GM Findlay, et al., Accurate classification of brca1 variants with saturation genome editing. *Nature* **562**, 217–222 (2018).
11. W Kabsch, C Sander, Dictionary of protein secondary structure: pattern recognition of hydrogen-bonded and geometrical features. *Biopolym. Orig. Res. on Biomol.* **22**, 2577–2637 (1983).
12. MZ Tien, AG Meyer, DK Sydykova, SJ Spielman, CO Wilke, Maximum allowed solvent accessibilites of residues in proteins. *PloS one* **8** (2013).
13. RA Laskowski, J Jabłońska, L Pravda, RS Vařeková, JM Thornton, Pdbsum: Structural summaries of pdb entries. *Protein science* **27**, 129–134 (2018).

14. PV Hornbeck, et al., Phosphositeplus, 2014: mutations, ptms and recalibrations. *Nucleic acids research* **43**, D512–D520 (2015).
15. K Wang, M Li, H Hakonarson, Annovar: functional annotation of genetic variants from high-throughput sequencing data. *Nucleic acids research* **38**, e164–e164 (2010).
16. H Mi, et al., Panther version 11: expanded annotation data from gene ontology and reactome pathways, and data analysis tool enhancements. *Nucleic acids research* **45**, D183–D189 (2017).
17. DR Zerbino, et al., Ensembl 2018. *Nucleic acids research* **46**, D754–D761 (2018).
18. GO Consortium, The gene ontology resource: 20 years and still going strong. *Nucleic acids research* **47**, D330–D338 (2019).
19. R Beaglehole, R Bonita, T Kjellström, , et al., *Basic epidemiology.* (World Health Organization Geneva), (1993).
20. A Viera, Odds ratios and risk ratios: what's the difference and why does it matter? *South. medical journal* **101**, 730–734 (2008).
21. SD Simon, Understanding the odds ratio and the relative risk. *J. andrology* **22**, 533–536 (2001).
22. PC Ng, S Henikoff, Sift: Predicting amino acid changes that affect protein function. *Nucleic acids research* **31**, 3812–3814 (2003).
23. IA Adzhubei, et al., A method and server for predicting damaging missense mutations. *Nat. methods* **7**, 248–249 (2010).
24. P Rentzsch, D Witten, GM Cooper, J Shendure, M Kircher, Cadd: predicting the deleteriousness of variants throughout the human genome. *Nucleic acids research* **47**, D886–D894 (2019).
25. O Wagih, et al., A resource of variant effect predictions of single nucleotide variants in model organisms. *Mol. systems biology* **14** (2018).
26. M Hicks, I Bartha, J di Iulio, JC Venter, A Telenti, Functional characterization of 3d protein structures informed by human genetic diversity. *Proc. Natl. Acad. Sci.* **116**, 8960–8965 (2019).
27. M Knudsen, C Wiuf, The cath database. *Hum. Genomics* **4**, 207–212 (2010).
